# Visible light photo-switching in a conformationally-strained electron acceptor *via* a dual singlet–triplet mechanism

**DOI:** 10.1039/d5sc03702f

**Published:** 2025-10-08

**Authors:** Sai Shruthi Murali, Aditi Kumar, Damon M. de Clercq, Céline Janiseck, Geoffrey R. Weal, Isabella Wagner, Kai Chen, Michael P. Nielsen, Timothy W. Schmidt, Justin M. Hodgkiss, Paul A. Hume

**Affiliations:** a School of Chemical and Physical Sciences, Victoria University of Wellington Wellington 6012 New Zealand paul.hume@vuw.ac.nz justin.hodgkiss@vuw.ac.nz; b School of Chemistry, UNSW Sydney Sydney NSW 2052 Australia; c School of Photovoltaic and Renewable Energy Engineering, UNSW Sydney Sydney NSW 2052 Australia; d MacDiarmid Institute for Advanced Materials and Nanotechnology Wellington 6012 New Zealand; e The Dodd-Walls Centre for Photonic and Quantum Technologies Dunedin 9016 New Zealand; f Robinson Research Institute, Faculty of Engineering, Victoria University of Wellington Wellington 6012 New Zealand

## Abstract

Molecular photo-switches have significant potential for use in smart materials that can be controlled by light. Photo-switches function due to lowering or complete removal of the barrier to switching in the excited state. This constraint on the ground and excited state potential energy surfaces means that relatively few photo-switching molecules are known, and fewer that only utilise visible photons. Here, we report the unanticipated visible-light photo switching behavior of a conformationally strained electron acceptor molecule, NIDCS-A, which consists of an anthracene core linked *via* vinylic double bonds to two thiophene-naphthalamide substituents. Using a combination of spectroscopic techniques, we find that NIDCS-A exhibits an unusual dual photo-switching mechanism *via* both singlet and triplet excited states. In the singlet state, photoisomerisation precedes geometric relaxation, which enables the molecule to overcome a surprisingly large energy barrier. At later times, photoisomerisation is mediated by long-lived triplet states. We draw several lessons for future work. First, photo-switching of vibrationally excited molecules is able to outcompete geometry relaxation. Second, our work reveals intrinsic molecular ISC as a complementary strategy to intramolecular triplet energy transfer for triplet-mediated photo-switching. Finally, we suggest that NIDCS-A may provide a template for the construction of all-optical three state molecular photo-switches.

## Abstract 2

A strained molecular electron acceptor undergoes visible light photo-switching *via* an unusual dual mechanism. Singlet isomerization proceeds *via* excess vibrational energy, while the triplet undergoes thermally activated isomerization.

## Graphical abstract

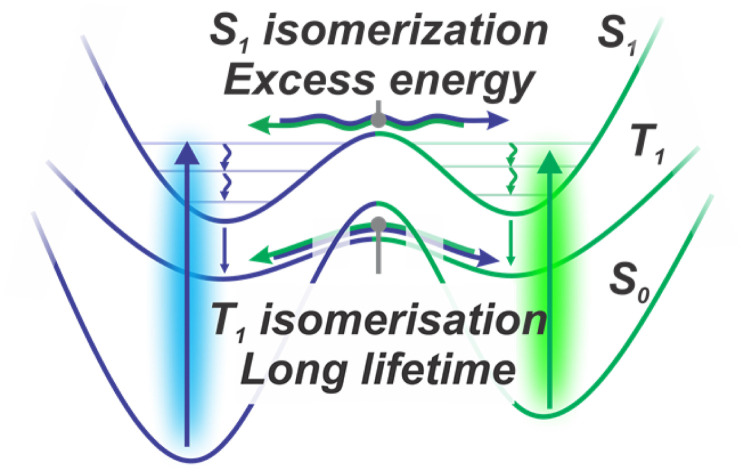

## Introduction

Photo-switches are molecules that can reversibly interconvert between two or more states through the absorption of light.^1,2^ This behaviour, combined with the fine spatial, temporal, and spectral precision of modern light sources, means that photo-switches show great promise to enable light-mediated control over a wide range of processes.^3^ Applications span fields as diverse as medicine, catalysis, and optoelectronics.^3–10^

Molecular photo-switches often require the use of high-energy UV photons to isomerise in at least one switching direction.^3,11–13^ This is problematic for materials science applications and in medicinal contexts, because of the damaging nature of UV photons and their low penetration depth in biological media.^3,11^ This has led to significant efforts to develop photo-switches that function using visible and/or NIR light.^3,11,12,14–18^ A range of creative optical tuning and energy transfer approaches have been explored,^3,7,12,17,19–21^ but an expansion of the space of visible/NIR-light photo-switching motifs is still needed.

**Fig. 1:**
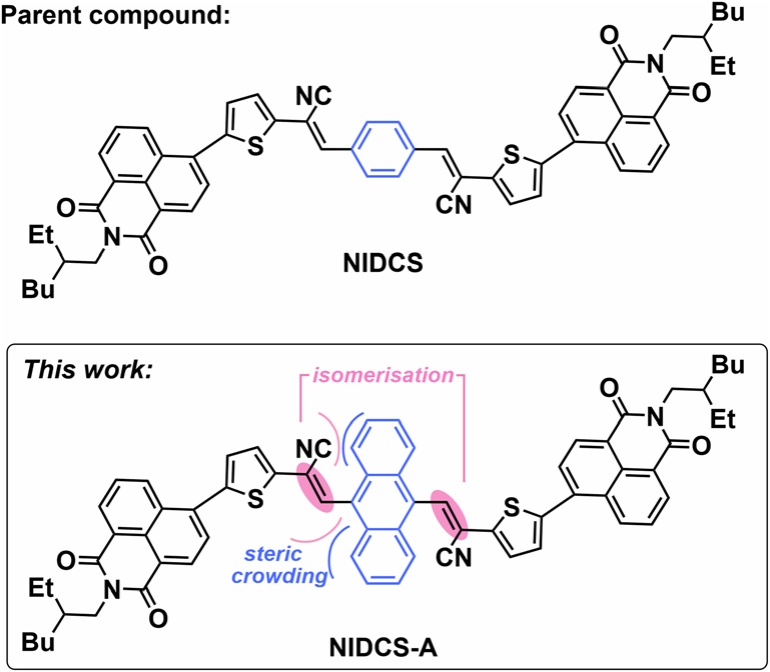
Molecular structure of NIDCS-A (reported herein) and the parent molecule, NIDCS.

Here, we describe the unanticipated visible light photo-switching of a novel geometrically strained molecular electron acceptor, NIDCS-A (Fig. 1).^22–24^ This compound was prepared following our recent work examining the role of intersystem crossing (ISC) in organic photovoltaic materials.^24^ However, unlike the other molecules in the NIDCS family, the geometric strain induced by the anthracene core in NIDCS-A induces wavelength-dependent photoisomerisation, leading to distinct isomeric compositions as a function of excitation wavelength. A combination of triplet sensitisation and quenching experiments reveal that the isomerisation process can be induced by triplet sensitisation but also occurs directly *via* the singlet excited state. Ultrafast spectroscopy and quantum chemical calculations reveal that photoisomerisation of NIDCS-A proceeds despite a significant energy barrier. The observed photo-switching behaviour is much faster than would be expected if relaxation of the molecular geometry were to precede isomerisation. It therefore appears that photo-switching is mediated by excess vibrational energy in the singlet excited state, in addition to proceeding thermally *via* long-lived triplet excited states. This bifurcating pathway is closest to that seen in diarylethylene, which switches between open and closed configurations *via* both singlets and ISC-derived triplets.^25^ The mechanism also shares similarity to intramolecular energy transfer systems^26–28^ and triplet-sensitisation of other well-known *E* ↔ *Z* photoswitches such as azobenzene and stilbene.^29–34^ Finally, we discuss the potential for NIDCS-A as a structural template for the design of three-state molecular switches driven by visible light.

## Results and discussion

### Photo-switching

**Fig. 2:**
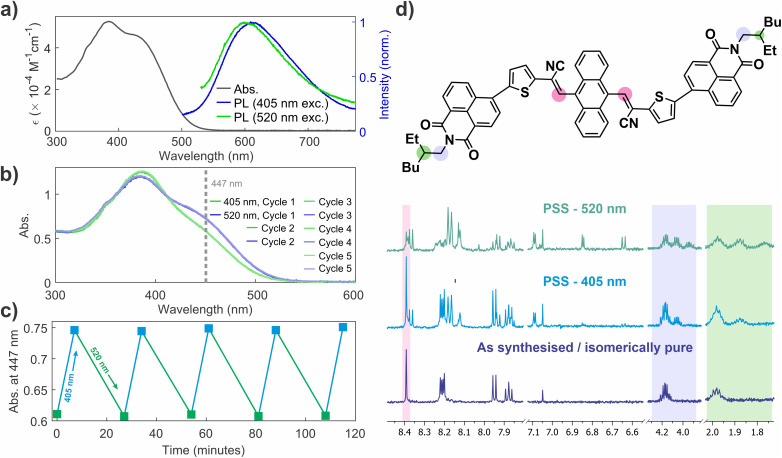
(a) Steady state UV-vis absorption spectra of NIDCS-A (10^−5^ M in dichloromethane) in isomerically pure form and photoluminescence spectra under 405 nm and 520 nm excitation. (b) UV-vis absorption spectra corresponding to photo-stationary states under 405 nm and 520 nm excitation, showing stability over 5 cycles. (c) Photo-switching monitored *via* absorbance at 447 nm under alternating 405 nm and 520 nm excitation. (d) Key changes in ^1^H NMR spectra of NIDCS-A in CDCl_3_ in isomerically pure form immediately following synthesis (bottom), photo-stationary state (PSS) under 405 nm photoexcitation (middle), and PSS under 520 nm excitation. Colored bands highlight diagnostic peaks associated with structural elements circled on the structure of NIDCS-A.

NIDCS-A was prepared *via* Knoevenagel condensation of anthracene-9,10-dialdehyde following the previously described synthetic sequence (see SI).^22,24^ The UV-vis absorption spectrum of freshly synthesised (isomerically pure) NIDCS-A shows a strong absorption band spanning 350–500 nm (Fig. 2a). Photoluminescence spectra under 405 nm and 520 nm excitation both lead to similar, broad emission peaked at ∼600 nm. The large Stokes shift of ∼180 nm points to significant geometric rearrangement in the excited state. The photoluminescence quantum efficiency (PLQE) was measured to be 2.1% under 405 nm excitation, and 1.8% under 520 nm.

The absorption spectrum of NIDCS-A was found to change upon exposure to ambient light, prompting an investigation of its optical properties. Fig. 2b shows the absorption spectra at photo-stationary states (PSS) formed upon 405 nm and 520 nm irradiation (PSS_405_ and PSS_520_, respectively). PSS_405_ and PSS_520_ have an isosbestic point at 411 nm that is not shared with the isomerically pure species in Fig. 2a. This indicates that PSS_405_ and PSS_520_ likely involve mixtures of more than two species, since the starting material is not completely consumed (see below). Switching between PSS_405_ and PSS_520_ is highly reversible, with no photodegradation observed over repeated excitation cycles (Fig. 2b and c).

NMR spectroscopy reveals that PSS_405_ and PSS_520_ correspond to different ratios of isomeric species (Fig. 2d). This suggests photoisomerisation of the two alkene double bonds to yield mixtures of *EE*, *EZ*/*ZE* and *ZZ* isomers (*c.f.*Fig. 1), in a similar manner to the Congo Red dianion.^35^ High resolution mass spectrometry showed no new *m*/*z* peaks following illumination, which also points towards photoisomerisation.

Specifically confirming alkene isomerisation is difficult in NIDCS-A due to the trisubstituted alkene linkages (*i.e.* neither the Karplus relation or NOESY correlations between vicinal hydrogens can be used), and the complex spectra of the photostationary mixtures. However, the NMR data support alkene isomerisation in several key ways. First, in the *EE* and *ZZ* isomers, the corresponding protons on the two substituents attached to anthracene are equivalent due to molecular symmetry. This symmetry is observed by NMR in the as-synthesised material, which we assign as the *EE* isomer. This conclusion is further supported by a NOESY correlation (Fig. S12) between the alkene proton resonance at 8.4 ppm and those of the closest anthracene protons at 7.65 ppm which is expected based on the DFT optimised geometry (see below). In the *EZ* isomer, the molecular symmetry is lost, which leads to two sets of resonances for the substituents. The unchanged *E* substituent is expected to exhibit peaks that are slightly shifted from their original positions (see Fig. S10), while the resonances from the *Z* substituent should be more significantly altered. This is the pattern seen in the NMR spectrum of PSS_405_, which we assign as a mixture of the *EE* and *EZ* isomers. This is also supported by NOESY spectroscopy. A new singlet resonance (*i.e.* alkene proton) associated with the isomerised substituent develops at 8.15 ppm. However, this resonance no longer shows correlations to the anthracene protons in the NOESY spectrum. This strongly suggests isomerisation, because the DFT-optimised geometry shows that the internuclear distance in the *E* configuration is 2.37 Å, while in the *Z* configuration, this distance increases to 2.75 Å, consistent with the loss of NOESY interaction. Similar features can be observed in the 2D spectra of PSS_520_.

We expect that PSS_520_ represents a mixture of *EE*, *EZ*, and *ZZ* isomers, but the complexity of the spectrum precludes a simple determination. This is in part because rotational isomerisation likely complicates the NMR analysis. Rotation around the single bonds directly linking the substituents to the anthracene core is expected to be relatively free when the substituents adopt an *E* configuration. But in the *ZZ* isomer rotation is prohibited due to steric interactions between the substituents and anthracene (*c.f.* DFT-calculated structures in Fig. 6). This means that rotamers placing the substituents on the same, or opposing, sides of the anthracene are possible, depending on the conformation during the isomerisation event. Nonetheless, the reversion of this mixture to the *EE* and *EZ* isomers under 405 nm excitation is strongly suggestive of alkene isomerisation.

### Sensitisation and quenching experiments show that singlet and triplet excited states can both mediate isomerisation

**Fig. 3:**
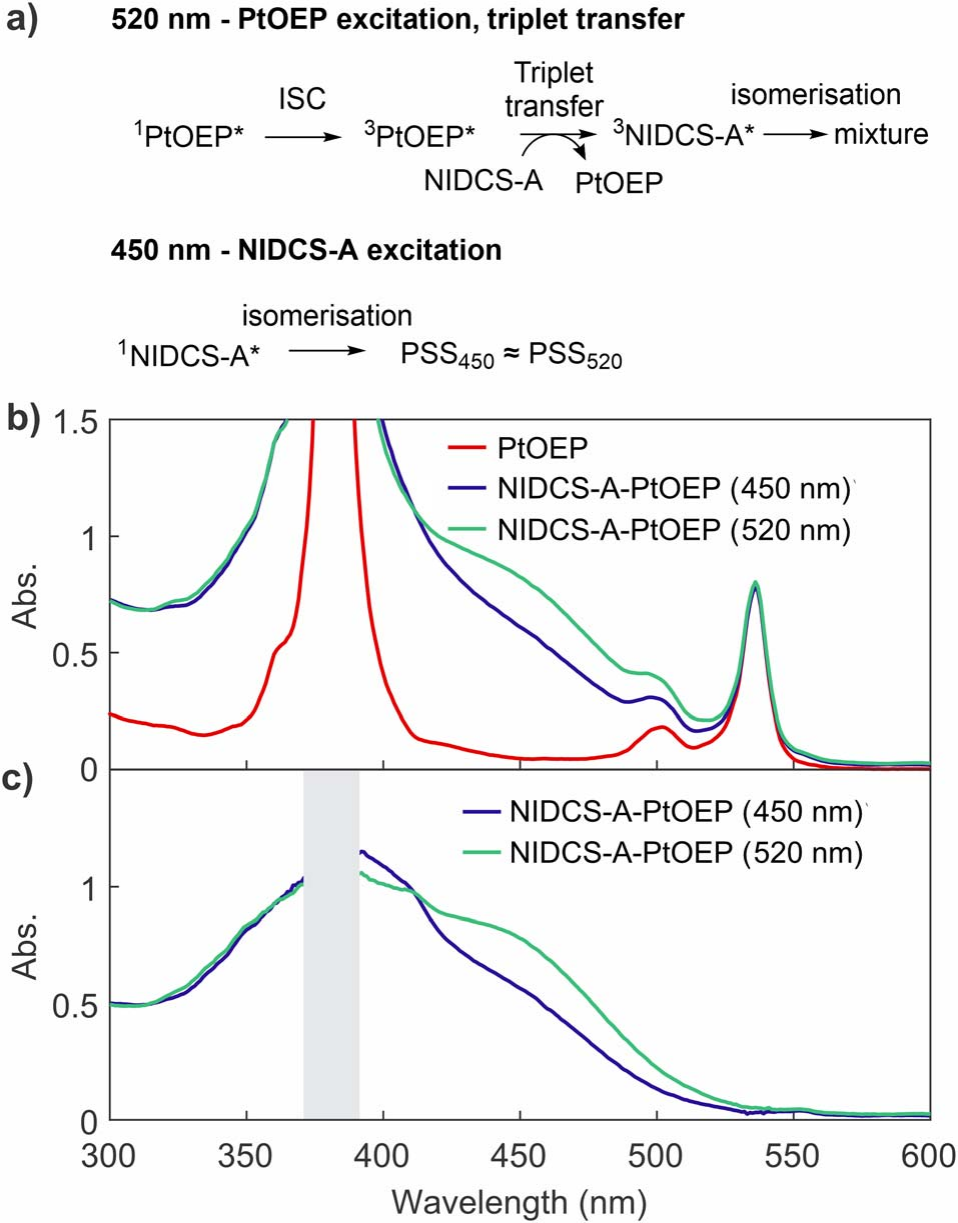
Photosensitisation pathway for photo-switching of NIDCS-A *via* excitation of platinum octaethylporphyrin (PtOEP), followed by triplet energy transfer. (a) Proposed reaction pathway. (b) UV-vis absorption spectra of a mixture of NIDCS-A (2 × 10^−5^ M) and PtOEP in dichloromethane, showing photo-stationary states under 450 nm and 520 nm excitation. An absorption spectrum of PtOEP normalised to match the peak at ∼535 nm is also shown for comparison. (c) ‘Pure’ NIDCS-A spectra obtained from those in part (b) by subtraction of the PtOEP spectrum. Grey bar covers a region where the spectral difference profile is compromised by strong absorption of the PtOEP Soret band.

To determine whether the triplet formation commonly observed in NIDCS materials was responsible for photo-switching, we performed triplet sensitisation experiments with platinum octaethyl porphyrin (PtOEP, Fig. 3a). Because PtOEP has significant absorption at both 405 nm and 520 nm, excitation at 405 nm was substituted for 450 nm, where PtOEP absorption is negligible. This ensures that we have one wavelength (450 nm) that only excites NIDCS-A and the other (520 nm) mainly excites the PtOEP.

Selective excitation of PtOEP at 520 nm induces switching of NIDCS-A. Importantly, the resulting PSS has a different absorption spectrum than when an NIDCS-A-only solution is excited at the same wavelength (PSS_520_). This behaviour is consistent with rapid ISC (^1^PtOEP* → ^3^PtOEP*) followed by collision-mediated triplet energy transfer to NIDCS-A molecules, which can then isomerise. Because triplet transfer is unlikely to be isomer selective, triplet-sensitised photo-switching results in a distinct isomeric distribution from PSS_520_ when PtOEP is not present. In contrast, when NIDCS-A is selectively excited at 450 nm, the absorption spectrum of the mixture resembles that of pure NIDCS-A excited at 520 nm (Fig. 3b and c). The reason for this resemblance is that for a pure NIDCS-A solution, no switching is observed under alternating 450 and 520 nm (*i.e.* PSS_450_ ≈ PSS_520_ for pure NIDCS-A).

While this experiment demonstrates that the triplet excited state of NIDCS-A can mediate isomerisation, it does not prove that triplet formation is necessary. We therefore examined the effect of two triplet quenchers on the photo-switching of NIDCS-A: oxygen (singlet energy 0.98 eV) and cyclooctatetraene (COT), which can accept triplets from molecules with T_1_ ≥ 0.8 eV.^36,37^ Interestingly, isomerisation is retained in the presence of both quenchers (Fig. S6 and S7). These experiments therefore bound the timescale over which T_1_-mediated photoisomerisation could be occurring, because any isomerisation *via* T_1_ would need to occur faster than the collision rate between NIDCS-A and quencher molecules. We find that photo-switching of NIDCS-A is still observed even when collisions with a triplet quencher occur every 20 ps (see the SI for detailed analysis). We therefore conclude that photoisomerisation *via* triplet states formed by ISC is an additional photo-switching mechanism for NIDCS-A, rather than the only pathway.

### Ultrafast spectroscopy reveals singlet photo-switching and triplet formation through ISC

**Fig. 4:**
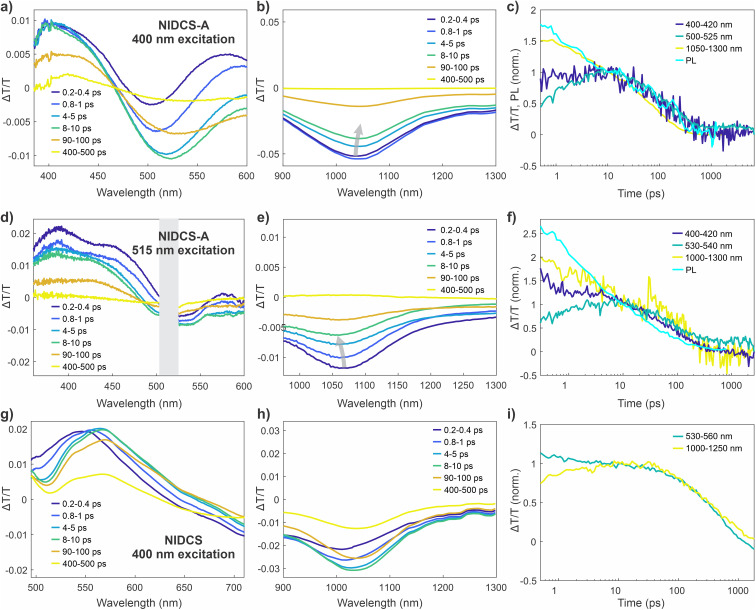
Transient absorption (TA) spectra of NIDCS-A solutions in dichloromethane compared with NIDCS solution (concentration ∼10^−4^ M). (a) Visible wavelength spectra of NIDCS-A, following excitation at 400 nm. (b) Near-IR wavelength spectra of NIDCS-A, following excitation at 400 nm. (c) TA kinetics of NIDCS-A and wavelength-integrated (500–650 nm) transient grating photoluminescence (PL) kinetics following excitation at 343 nm, normalised to signal intensity at 10 ps. (d) Visible wavelength spectra of NIDCS-A, following excitation at 515 nm. Grey bar covers a region of data where the spectral shape is compromised by 515 nm pump scatter. (e) Near-IR wavelength spectra of NIDCS-A, following excitation at 515 nm. (f) TA kinetics of NIDCS-A following excitation at 515 nm, and wavelength-integrated (550–650 nm) transient grating PL kinetics following excitation at 343 nm, normalised to signal intensity at 10 ps. (g) Visible wavelength spectra of NIDCS, following excitation at 400 nm. (h) Near-IR wavelength spectra of NIDCS following excitation at 400 nm. (i) TA kinetics of NIDCS following excitation at 400 nm, normalised to signal intensity at 10 ps.

To better understand the photo-switching behaviour of NIDCS-A, we performed a series of ultrafast transient absorption (TA) and transient grating photoluminescence (TGPL) measurements. In TA measurements (Fig. 4a–f), the initially formed NIDCS-A singlet exhibits a positive (Δ*T*/*T* > 0) ground state bleach (GSB) at 400–500 nm and stimulated emission (SE) at 520–600 nm, which resemble the steady state absorption and photoluminescence spectra, respectively. The singlet is also characterised by a broad negative (Δ*T*/*T* < 0) photoinduced absorption (PIA) at ∼1050 nm (Fig. 4b and e). Different TA spectra are observed using different excitation wavelengths. The GSB shapes confirm that distinct subsets of NIDCS-A isomers are photo-selected by the two pump wavelengths. Excitation at 400 nm gives a GSB closer to the absorption spectrum of PSS_520_ while the GSB under 515 nm excitation resembles the absorption profile of the freshly synthesised NIDCS-A (Fig. 4d and e). Excitation at 400 nm also leads to stronger SE and PIA features compared to excitation at 515 nm, reflecting the differences in the excited state populations generated under different wavelength excitation. The singlet PIA (1000–1100 nm) tracks the isomerisation: a dynamic red shift of the peak is observed when exciting with 400 nm, while a blue shift is observed when exciting with 515 nm. These shifts suggest that interconversion between isomeric species occurs over the timescale of these experiments (*i.e.* within the singlet lifetime).

The TA spectra of NIDCS-A exhibit pronounced early picosecond dynamics.^23,24^ Significant spectral evolution occurs within 10–20 ps with decay of the SE accompanied by the development of a negative signal at 500–550 nm. This change is due to an underlying PIA combined with dynamic red shifting of the SE from ∼560 nm to ∼600 nm. After this time, the excited state absorption spectrum attains a relatively steady shape, with similar decay kinetics at all probe wavelengths. From ∼100 ps onwards, the appearance of a long-lived species becomes apparent. In this new state, the GSB remains, but the singlet PIA at 1050 nm and SE peak at 600 nm are replaced by a new PIA centred at 530 nm. Intensity dependent measurements confirmed that all kinetics are not fluence dependent (SI, Fig. S14), which means that aggregation effects are not responsible for the dynamics.

The fluorescence dynamics probed by TGPL spectroscopy (overlaid on Fig. 4c) closely match those of the singlet PIA at 1050 nm from the TA measurements. This confirms that the excited state population is dominated by emissive singlet excited states for the first ∼100 ps after photoexcitation. The non-emissive species remaining at late time is therefore assigned as the NIDCS-A triplet, formed *via* ISC. This assignment is motivated by the spectral similarity to triplets in other NIDCS derivatives^24^ and is also supported by triplet sensitisation experiments (see Fig. S16). Similar amounts of triplets are formed regardless of excitation wavelength (Fig. S13), with an estimated triplet yield of 27% as determined by triplet sensitisation experiments (see SI). Importantly, while the singlet and triplet TA spectra of NIDCS-A resemble those of the parent compound NIDCS (provided for comparison in Fig. 4g–i) and other analogues, the early picosecond dynamics are not observed in other members of the NIDCS family.^24^ This also suggests a connection between the photoisomerisation and the structural rearrangements observed *via* TA.

**Fig. 5:**
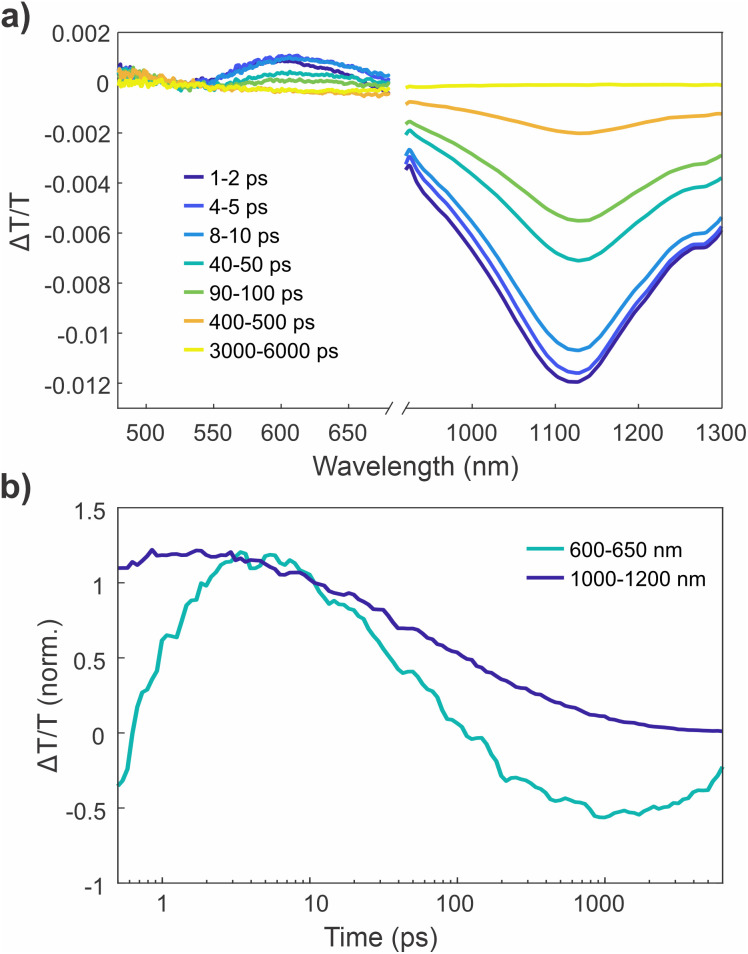
Ultrafast transient absorption spectra and kinetics of an NIDCS-A film. (a) TA spectra (400 nm, 100 fs pump pulse), showing the evolution of spectra from singlet (blue) to triplet (yellow). (b) Kinetics at different regions normalized at 0.5 ps.

TA measurements of NIDCS-A thin films exhibit key differences with the solution measurements that are consistent with suppressed photoisomerisation (Fig. 5). The spectral features in the film state are initially similar to the solution measurements. However, the SE in the film grows and red shifts over the first 1–2 ps, without the development of the strong overlapping PIA that is seen in the solution measurements. This change in photophysical behaviour strongly suggests that the dynamics observed in solution involve significant changes in molecular geometry, that are suppressed in the rigid environment of a solid-state film. That is, photoisomerisation occurs in solution, while the film is characterised by a more typical geometry relaxation/planarisation process. As with the solution measurements and other compounds in the NIDCS series, a small triplet population is observed at later time delays.

In summary, we interpret our ultrafast spectroscopy results as follows. In solution, photoexcitation populates the S_1_ state, which undergoes a *E*/*Z* photoisomerisation over 10–20 ps. These dynamics are absent in other members of the NIDCS family and are suppressed in the solid state, which is consistent with photoisomerisation. We also observe triplet formation *via* ISC in NIDCS-A, likely mediated by conformational disorder and possibly also the anthracene core. The triplet sensitisation measurements in the previous section show that these triplets also mediate isomerisation.

### DFT calculations

**Fig. 6:**
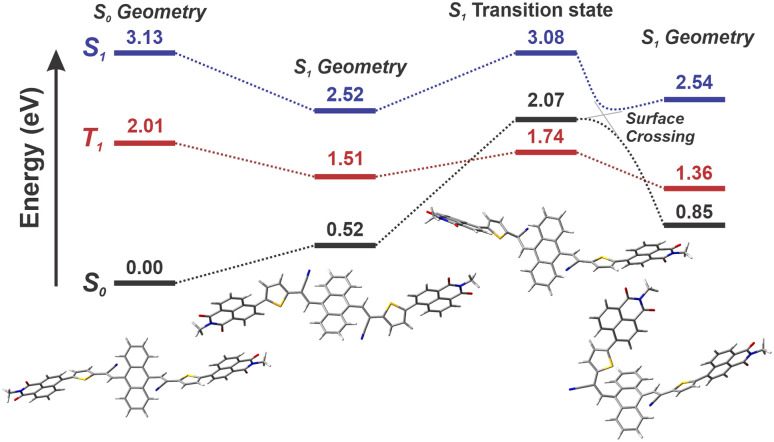
TD-DFT-calculated singlet/triplet energy levels for NIDCS-A. Calculations were performed using the CAM-B3LYP exchange–correlation functional, 6-31G(d,p) basis set, and polarisable continuum solvation with the parameters of CH_2_Cl_2_.^39,40^ Excited state calculations were performed using the Tamm–Dankoff approximation.

To understand the mechanism of photoisomerisation, we examined the potential energy surfaces for *E*/*Z* alkene photoisomerisation (Fig. 6). In S_0_, DFT calculations yield an insurmountable barrier at room temperature, with relaxed potential energy scans of the alkene dihedral angle giving an estimated activation energy of ∼50 kcal mol^−1^. These calculations were unable to yield a smooth trajectory due to sudden changes in geometry as the molecule passes close to the transition state, even with a small step size of 1°. In S_1_, a transition state corresponding to a barrier of 9.2 kcal mol^−1^ was found. Propagation of the molecular geometry along the intrinsic reaction coordinate starting from this transition state leads to an apparent avoided surface crossing with S_0_. Potential energy scans were also performed for the parent compound, NIDCS (*c.f.*Fig. 1). These revealed a significantly increased isomerisation barrier in S_1_ of ∼15 kcal mol^−1^, consistent with the lack of photo-switching observed for the other compounds in the NIDCS family. That is, the strain introduced by the bulky anthracene core prevents planarisation and destabilises the molecule, lowering the energy barrier to switching.

The isomerisation barrier height of 9.2 kcal mol^−1^ for NIDCS-A corresponds to a predicted rate of 1.2 × 10^6^ s^−1^ according to the Eyring equation,^38^ which means that thermally activated photo-switching cannot explain the spectral evolution occurring in the first 10–20 ps. Combining an isomerisation rate constant of 1.2 × 10^6^ s^−1^ with a singlet lifetime of ∼100 ps yields an estimated quantum yield of 0.02% in the S_1_ state. Under the laser power used for our photo-switching experiments (11.4 mW at 405 nm), this would yield a maximum of ∼2.3 × 10^−10^ mol successful reactions within 10 minutes, which is negligible compared to the number of NIDCS-A molecules in solution (∼10^−7^ mol).

These considerations inform the mechanism of photo-switching. Our triplet quenching and ultrafast spectroscopy experiments show that photoisomerisation occurs in the singlet state but is accelerated over what would be expected based on DFT if the molecule is assumed to undergo geometric relaxation in the excited state prior to isomerisation. We therefore conclude that excess vibrational energy following photoexcitation enables NIDCS-A to cross the isomerisation barrier before vibrational relaxation is complete. This hypothesis is consistent with the large Stokes shift (1.2 eV for NIDCS-A *c.f.* 0.6 eV for NIDCS), the pronounced early time dynamics observed by TA, and DFT calculations showing significant molecular reorganisation following excitation to S_1_. In the triplet excited state, there is more time for photoisomerisation to occur as the triplet lifetime is expected to be on the ns–μs timescale, and a higher isomerisation yield is therefore expected. Though the probability of forming a triplet is ∼27% for a given singlet, over multiple excitation events many molecules eventually form triplets and can isomerise *via* this pathway.

Another question is whether a single photoexcitation is capable of inducing isomerisation of both substituents, in a similar manner to the multi-step switching mechanism of donor–acceptor Stenhouse adducts.^41,42^ In the case of DA Stenhouse adducts, the initial alkene isomerisation is photochemically driven, followed by thermal rotation and electrocyclisation.^41,42^ This is different to NIDCS-A, where two non-thermal alkene isomerisation events are required. Based on our DFT calculations, double switching appears unlikely in the S_1_ state. The presence of an avoided crossing along the isomerisation coordinate means that molecules that isomerise are also likely to undergo rapid decay to the ground state, necessitating absorption of a second photon for isomerisation of the remaining substituent. However, double switching may indeed be possible in the triplet state. The best way to test this question experimentally would be to use ion mobility mass spectrometry on a charged analogue^35,43^ of NIDCS-A, which is suggested as an avenue for future work.

### Discussion

**Fig. 7:**
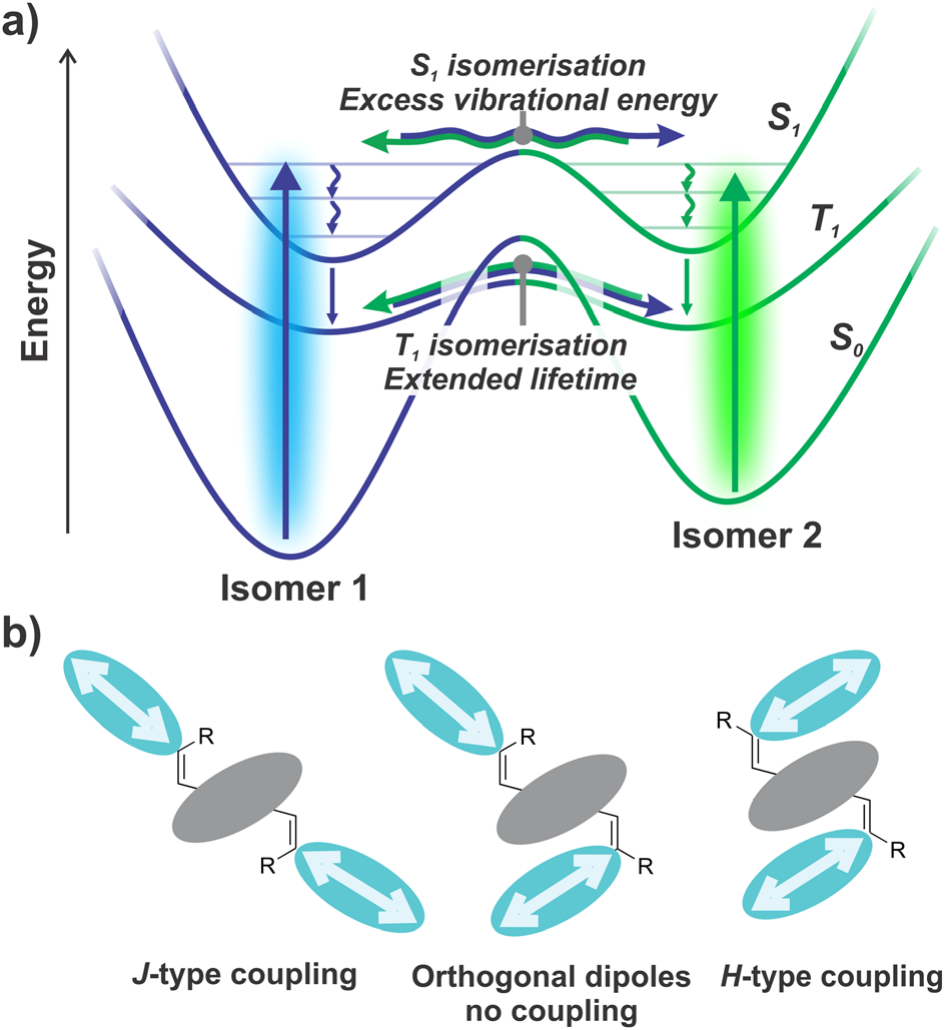
(a) Photo-isomerisation using excess vibrational energy to overcome an appreciable barrier in the excited state. (b) Concept for three-state molecular photo-switches using geometry-dependent interactions between transition dipole moments.

The photo-switching of NIDCS-A suggests several interesting pathways for further exploration. First, the ability of this molecule to overcome an appreciable energy barrier in less than a nanosecond by using excess vibrational energy may enable photo-switching of a wider variety of structures. Perhaps, by designing molecules to undergo large structural changes in the excited state, large barriers to photoisomerisation can be overcome by enabling isomerisation to outcompete geometric relaxation (Fig. 7a). These large amplitude nuclear motions, which are reflected in the low PLQE (∼2%), may also be a key driver of the significant ISC yield (27%) as found in other NIDCS-based materials.^24^ That is, structural deformation appears to assist photoswitching *via* both singlet and triplet pathways.

Second, with its three possible configurational isomers, NIDCS-A may provide a useful structural template for the construction of three state optical switches. By replacing the terminal naphthalene diimide end groups with more sophisticated chromophores, it should be possible to extend the absorption further into the visible spectrum. Both electronic and excitonic interactions between the chromophores are expected to be highly sensitive to the molecular geometry, which may lead to differences in absorption profile that can be utilised for photo-switching (Fig. 7b). For example, a colinear J-type aggregate would be expected to yield red-shifted absorption and increase intensity of the 0–0 vibronic transition relative to uncoupled chromophores.^44^ Conversely, a cofacial H-type aggregate would show blue-shifted absorption, with increased intensity of the 0–1 vibronic transition.

Finally, although triplets are not needed for photoisomerisation in NIDCS-A, the use of ISC to extend excited state lifetimes through the formation of triplets still appears to be a promising avenue for investigation. Recent years have seen considerable success in studies of triplet-sensitised photo-switching, including intramolecular variants. The present study suggests that ISC mediated by conformational motion of motifs such as thiophene may also be a useful way to initiate photo-switching, without the need for a traditional triplet sensitising unit.

## Conclusions

A conformationally strained electron acceptor material NIDCS-A was synthesised and found to function as a visible light photo-switch. A combination of spectroscopic experiments and theoretical calculations showed significant structural changes occurring within the singlet lifetime, and the formation of triplets through ISC. In the singlet excited state, photoisomerisation proceeds in S_1_ due to the excess vibrational energy available immediately following light absorption. Triplet sensitisation experiments that photoisomerisation can occur *via* the triplet excited state, while studies using triplet quenchers (O_2_ and cyclooctatetraene) demonstrated that triplets are not required for photo-switching upon direct photoexcitation. This unusual dual mode photoisomerisation mechanism suggests that structural relaxation and ISC may be useful effects to inform the design of unconventional and three state molecular photoswitches.

## Author contributions

S. S. M. synthesised materials, performed steady-state optical and electrochemical characterisation, and ultrafast transient absorption spectroscopy measurements. A. K. performed steady-state optical characterization, including photo-switching, triplet-quenching, and sensitization experiments, as well as steady-state photoluminescence and absolute photoluminescence quantum yield measurements. D. M. d. C. performed ultrafast and nanosecond transient absorption spectroscopy measurements. C. J. assisted with the development of the synthetic methodology. G. W. assisted with theoretical calculations. I. W. performed the transient grating photoluminescence measurements. K. C. supervised I. W. and assisted with transient absorption measurements. M. P. N., T. W. S., K. C., and J. M. H. supervised the TA experiments and provided feedback on the draft manuscript. P. A. H. conceived the project, supervised the synthesis, characterisation, and ultrafast spectroscopy measurements, and performed theoretical calculations. All authors discussed the results and wrote the paper.

## Conflicts of interest

There are no conflicts to declare.

## Supplementary Material

SC-016-D5SC03702F-s001

## Data Availability

The experimental and computational data associated with this article have been provided in the document of supplementary information (SI). Supplementary information: synthetic methodology, optical and electrochemical characterization data, triplet sensitization and quenching experiments, and details of theoretical calculations. See DOI: https://doi.org/10.1039/d5sc03702f.

## References

[cit1] Goulet-Hanssens A., Eisenreich F., Hecht S. (2020). Adv. Mater..

[cit2] Feringa B. L. and Browne W. R., Molecular Switches, Wiley-VCH, Weinhem, 2nd edn, 2011

[cit3] Zhang Z., Wang W., O'Hagan M., Dai J., Zhang J., Tian H. (2022). Angew. Chem., Int. Ed..

[cit4] Cheng H.-B., Zhang S., Bai E., Cao X., Wang J., Qi J., Liu J., Zhao J., Zhang L., Yoon J. (2022). Adv. Mater..

[cit5] Xu F., Feringa B. L. (2023). Adv. Mater..

[cit6] Lubbe A. S., Szymanski W., Feringa B. L. (2017). Chem. Soc. Rev..

[cit7] Irie M., Fukaminato T., Matsuda K., Kobatake S. (2014). Chem. Rev..

[cit8] Klajn R. (2014). Chem. Soc. Rev..

[cit9] Volarić J., Szymanski W., Simeth N. A., Feringa B. L. (2021). Chem. Soc. Rev..

[cit10] Ishow E., Brosseau A., Clavier G., Nakatani K., Pansu R. B., Vachon J. J., Tauc P., Chauvat D., Mendonça C. R., Piovesan E. (2007). J. Am. Chem. Soc..

[cit11] Xu F., Sheng J., Stindt C. N., Crespi S., Danowski W., Hilbers M. F., Buma W. J., Feringa B. L. (2024). Chem. Sci..

[cit12] Bléger D., Schwarz J., Brouwer A. M., Hecht S. (2012). J. Am. Chem. Soc..

[cit13] Kuntze K., Isokuortti J., van der Wal J. J., Laaksonen T., Crespi S., Durandin N. A., Priimagi A. (2024). Chem. Sci..

[cit14] Lameijer L. N., Budzak S., Simeth N. A., Hansen M. J., Feringa B. L., Jacquemin D., Szymanski W. (2020). Angew. Chem., Int. Ed..

[cit15] Köttner L., Ciekalski E., Dube H. (2023). Angew. Chem., Int. Ed..

[cit16] Petermayer C., Thumser S., Kink F., Mayer P., Dube H. (2017). J. Am. Chem. Soc..

[cit17] Bléger D., Hecht S. (2015). Angew. Chem., Int. Ed..

[cit18] Leistner A. L., Pianowski Z. L. (2022). Eur. J. Org. Chem..

[cit19] Crespi S., Simeth N. A., König B. (2019). Nat. Rev. Chem..

[cit20] Bandara H. M. D., Burdette S. C. (2012). Chem. Soc. Rev..

[cit21] Pfeifer L., Scherübl M., Fellert M., Danowski W., Cheng J., Pol J., Feringa B. L. (2019). Chem. Sci..

[cit22] Kwon O. K., Park J.-H., Park S. K., Park S. Y. (2015). Adv. Energy Mater..

[cit23] Shi J., Isakova A., Abudulimu A., van den Berg M., Kwon O. K., Meixner A. J., Park S. Y., Zhang D., Gierschner J., Lüer L. (2018). Energy Environ. Sci..

[cit24] Murali S. S., Gallaher J. K., Janiseck C., Tay E. J., Wagner I., Thorn K. E., Ilina A., Tamming R. R., Wang J., Sester C., Sutton J. J., Price M. B., Gordon K. C., Chen K., Zhan X., Hodgkiss J. M., Hume P. A. (2023). J. Am. Chem. Soc..

[cit25] Indelli M. T., Carli S., Ghirotti M., Chiorboli C., Ravaglia M., Garavelli M., Scandola F. (2008). J. Am. Chem. Soc..

[cit26] Irie M., Fukaminato T., Matsuda K., Kobatake S. (2014). Chem. Rev..

[cit27] Ikariko I., Kim S., Hiroyasu Y., Higashiguchi K., Matsuda K., Hirose T., Sotome H., Miyasaka H., Yokojima S., Irie M., Kurihara S., Fukaminato T. (2022). J. Phys. Chem. Lett..

[cit28] Chen K., Liu J., Andréasson J., Albinsson B., Liu T., Hou L. (2024). Chem. Sci..

[cit29] Larsson W., Morimoto M., Irie M., Andréasson J., Albinsson B. (2023). Chem.–Eur. J..

[cit30] Kuntze K., Isokuortti J., van der Wal J. J., Laaksonen T., Crespi S., Durandin N. A., Priimagi A. (2024). Chem. Sci..

[cit31] Li Z., Zeng X., Gao C., Song J., He F., He T., Guo H., Yin J. (2023). Coord. Chem. Rev..

[cit32] Morikawa M., Mizuno M., Harada N., Kimizuka N. (2023). Chem. Lett..

[cit33] Isokuortti J., Kuntze K., Virkki M., Ahmed Z., Vuorimaa-Laukkanen E., Filatov M. A., Turshatov A., Laaksonen T., Priimagi A., Durandin N. A. (2021). Chem. Sci..

[cit34] Neveselý T., Wienhold M., Molloy J. J., Gilmour R. (2022). Chem. Rev..

[cit35] Bull J. N., Scholz M. S., Carrascosa E., da Silva G., Bieske E. J. (2018). Phys. Rev. Lett..

[cit36] Schols S., Kadashchuk A., Heremans P., Helfer A., Scherf U. (2009). ChemPhysChem.

[cit37] Mai V. T. N., Ahmad V., Mamada M., Fukunaga T., Shukla A., Sobus J., Krishnan G., Moore E. G., Andersson G. G., Adachi C., Namdas E. B., Lo S. C. (2020). Nat. Commun..

[cit38] Eyring H. (1935). J. Chem. Phys..

[cit39] Yanai T., Tew D. P., Handy N. C. (2004). Chem. Phys. Lett..

[cit40] Frisch M. J. , Trucks G. W., Schlegel H. B., Scuseria G. E., Robb M. A., Cheeseman J. R., Scalmani G., Barone V., Petersson G. A., Nakatsuji H., Li X., Caricato M., Marenich A. V., Bloino J., Janesko B. G., Gomperts R., Mennucci B., Hratchian H. P., Ortiz J. V., Izmaylov A. F., Sonnenberg J. L., Williams-Young D., Ding F., Lipparini F., Egidi F., Goings J., Peng B., Petrone A., Henderson T., Ranasinghe D., Zakrzewski V. G., Gao J., Rega N., Zheng G., Liang W., Hada M., Ehara M., Toyota K., Fukuda R., Hasegawa J., Ishida M., Nakajima T., Honda Y., Kitao O., Nakai H., Vreven T., Throssell K., Montgomery Jr J. A., Peralta J. E., Ogliaro F., Bearpark M. J., Heyd J. J., Brothers E. N., Kudin K. N., Staroverov V. N., Keith T. A., Kobayashi R., Normand J., Raghavachari K., Rendell A. P., Burant J. C., Iyengar S. S., Tomasi J., Cossi M., Millam J. M., Klene M., Adamo C., Cammi R., Ochterski J. W., Martin R. L., Morokuma K., Farkas O., Foresman J. B., and Fox D. J., Gaussian 16, Revision C.01, Gaussian, Inc., Wallingford CT, 2016

[cit41] Di Donato M., Lerch M. M., Lapini A., Laurent A. D., Iagatti A., Bussotti L., Ihrig S. P., Medved' M., Jacquemin D., Szymański W., Buma W. J., Foggi P., Feringa B. L. (2017). J. Am. Chem. Soc..

[cit42] Zulfikri H., Koenis M. A. J., Lerch M. M., Di Donato M., Szymański W., Filippi C., Feringa B. L., Buma W. J. (2019). J. Am. Chem. Soc..

[cit43] Bull J. N., Carrascosa E., Mallo N., Scholz M. S., da Silva G., Beves J. E., Bieske E. J. (2018). J. Phys. Chem. Lett..

[cit44] Spano F. C. (2010). Acc. Chem. Res..

