# Supplementary material for: Visible light photo-switching in a conformationally-strained electron acceptor via a dual singlet–triplet mechanism
Source: Chem Sci. 2025 Oct 8;16(45):21489–97. doi: 10.1039/d5sc03702f (PMC12522393; doi:10.1039/d5sc03702f)
Supplement: SC-016-D5SC03702F-s001 [file SC-016-D5SC03702F-s001.pdf]

Supplementary Information (SI) for Chemical Science.
This journal is © The Royal Society of Chemistry 2025

Supporting Information

1. Synthesis and Characterisation of NIDCS-A

1.1. General Information

Commercially available chemicals were used without further purification. Anhydrous solvents were obtained according to literature methods and stored over molecular sieves. Analytical thin layer chromatography was performed with Merck TLC Silica gel F254 coated on aluminium sheets. Proton nuclear magnetic resonance spectra (^{1}H NMR) were recorded on a JEOL JMTC-500/54/JJ NMR spectrometer. Chemical shifts for ^{1}H NMR spectra are reported as in units of parts per million (ppm) downfield from $SiMe_4$ (δ 0.0). Multiplicities are reported as: s (singlet), d (doublet), t (triplet), q (quartet), dd (doublet of doublets), or m (multiplet). Coupling constants *J* are reported in Hz. Chemical shifts of carbon nuclear magnetic resonance spectra (^{13}C NMR) are reported as δ in units of parts per million (ppm) downfield from $SiMe_4$ (δ 0.0). High-resolution mass spectra were recorded using Agilent 6530 LCMS.

1.2. Synthetic Procedure for NIDCS-A

NIDCS-A was synthesised by Knoevenagel condensation of 9,10 anthracene dialdehyde with NI precursor by adapting literature procedures.[1–3]

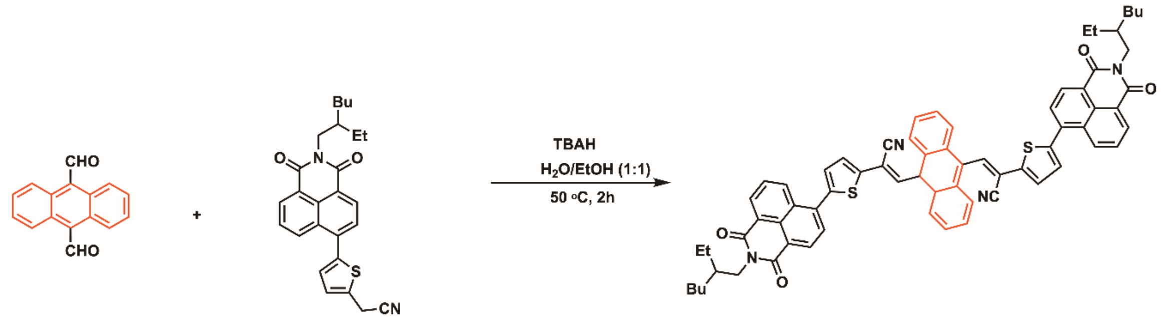

1 2 3

Figure S1. Knoevenagel condensation of dialdehyde with NI precursor.

NIDCS-A / (2*E*,2'*E*)-3,3'-(Anthracene-9,10-diyl)bis(2-(5-(2-(2-ethylhexyl)-1,3-dioxo- 2,3-dihydro-1H-benzo[de]isoquinolin-6-yl)thiophen-2-yl)acrylonitrile)

To a stirred solution of 9,10 anthracene dialdehyde (1 equivalents) and compound 2 (2.3 equivalents) in EtOH/H_2O (1:1,10mL) at 50°C was added tetrabutylammonium hydroxide (1 M in MeOH, 0.1 equivalents). After two hours, the reaction was cooled to room temperature and quenched by the addition of aq. HCl (10 mL, 5 M). The resultant precipitate was collected by filtration and washed with water. The filtrate was then dispersed in diethyl ether and the remaining solid was collected by filtration. Recrystallization from ethyl acetate afforded the product as a coloured powder.

NIDCS-A / (2*E*,2'*E*)-3,3'-(Anthracene-9,10-diyl)bis(2-(5-(2-(2-ethylhexyl)-1,3-dioxo- 2,3-dihydro-1H-benzo[de]isoquinolin-6-yl)thiophen-2-yl)acrylonitrile)

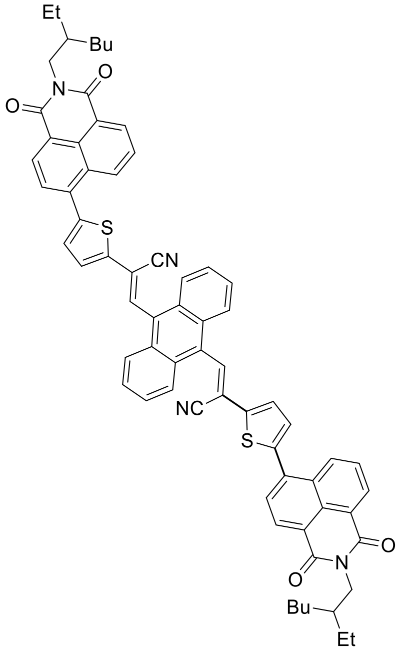

Yield: 25% (28 mg, 0.03 mmol).

Physical Appearance: Orange powder.

Melting Point: > 260°C.

^{1}H NMR (500 MHz, $CDCl_3$, TMS): *δ* 8.72-8.70 (m, 4H), 8.68 (d, *J*= 7.5 *Hz*, 2H), 8.39 (s, 2H), 8.21 (dd, J_1 = 6.5 *Hz*, J_2= 3.0 *Hz,* 4H), 7.95 (d, *J*= 7.5 *Hz*, 2H), 7.87 (t, *J*= 8.0 *Hz*, 2H), 7.66-7.67 (m, 4H) 7.66 (d, *J* = 4.0 *Hz*, 2H), 7.43 (d, *J*= 4.0 *Hz*, 2H), 4.12-4.21 (m, 4H), 1.96 1.99 (m, 2H), 1.37-1.42 (m, 8H), 1.32- 1.36 (m, 8H), 0.96 (t, *J*= 7.5 *Hz*, 6H), 0.90 (t, *J* = 7.0 *Hz*, 6H) ppm.

^{13}C NMR (126 MHz, $CDCl_3$, TMS): *δ* 164.6 (2×C=O), 164.3 (2×C=O), 141.9 (2×C), 139.5 (2×C), 138.0 (2×Vinyl-CH), 137.6 (2×C), 131.8 (4×ArCH), 130.8 (2×ArCH), 130.0 (2×ArCH + 2×C), 129.8 (4×C), 129.4 (4×C), 129.0 (2×C), 128.9 (2×ArCH), 127.8 (2×ArCH), 127.2 (4×ArCH), 126.0 (4×ArCH), 123.4 (2×C), 123.0 (2×C), 115.9 (2×C), 115.1 (2×C), 44.4 (2×CH_2), 38.1 (2×CH), 30.9 (2×CH_2), 28.9 (2×CH_2), 24.2 (2×CH_2), 23.2 (2×CH_2), 14.3 (2×CH_3), 10.8 (2×CH_3) ppm.

IR: ν_{max} 2952, 2926, 2857, 2224, 1696, 1654, 1585, 1438, 1380, 1350, 1232, 1183, 1098, 1030, 899, 854, 776, 735, 449 cm^{-1}.

UV-Vis: λ_{max} = 385 nm,
ε_{max} = 5.26×10^4 M^{-1} cm^{-1}

HRMS (ESI): Calcd M^{+} for $C_{68}H_{58}N_4O_4S_2$: 1059.3933, found: 1059.3974.

1.3. NMR Data

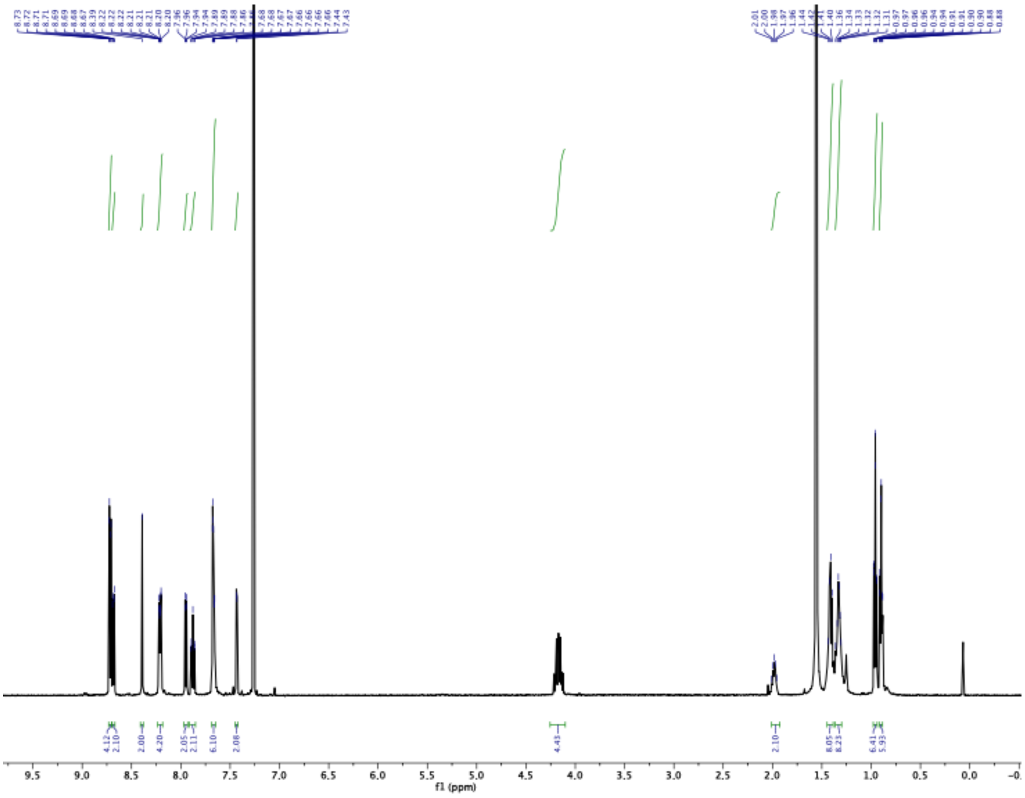

Figure S2. ^{1}H NMR of NIDCS-A in $CDCl_3$.

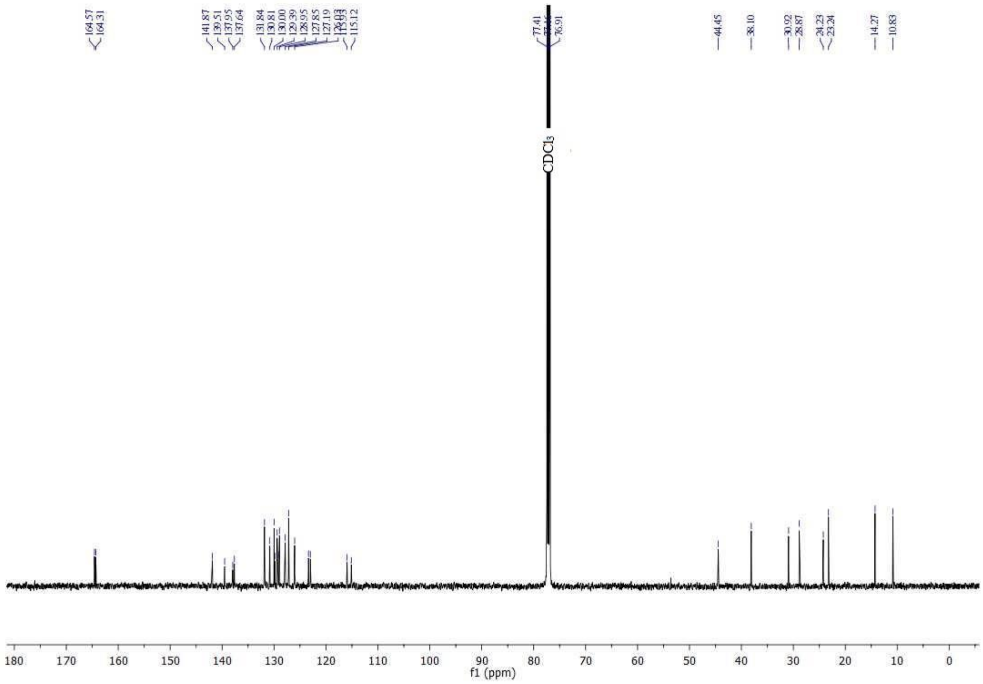

Figure S3. ^{13}C NMR of NIDCS-A in $CDCl_3$.

1.4. Cyclic Voltammetry

The LUMO energy of NIDCS-A was estimated from cyclic voltammetry, while the HOMO level could not be estimated accurately due to non-reversibility of the oxidation process. CV measurements employed a three-electrode assembly: a glassy carbon working electrode, platinum counter electrode, and silver wire pseudo-reference. Measurements were carried out in a single compartment cell containing 0.2 mM NIDCS-A in DCM solution with tetrabutylammonium hexafluorophosphate (Bu_4NPF_6) as the supporting electrolyte at a scan rate of 100 mV s^{-1}. The ferrocene/ferrocene$^+$ (Fc/Fc^+) redox couple was used as an internal standard to reference the reduction potential of NIDCS-A. Specifically, the LUMO energy level was calculated according to:

$$E_{LUMO} = -(E_{red}-E_{1/2\ Fc/Fc+} + 4.8)\ \text{eV}$$

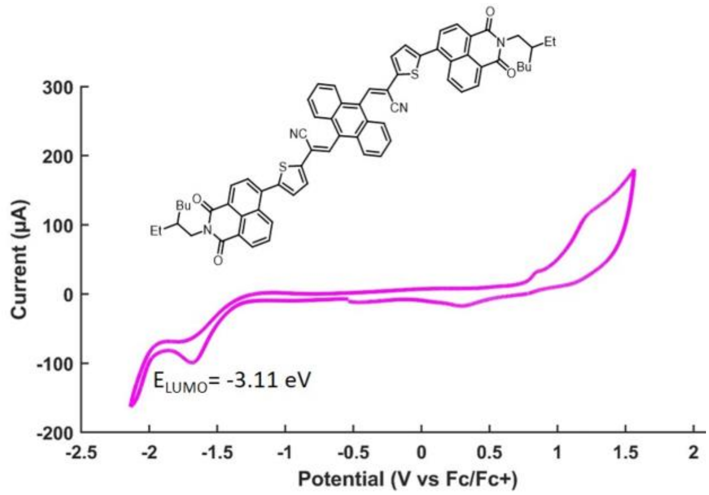

Figure S4. Cyclic voltammogram of NIDCS-A in DCM/0.1M Bu_4NPF_6 obtained with a scan rate of 100mV/s.

2. Steady State Optical Spectroscopy

2.1. General Procedures

UV-Vis absorption spectra and photoluminescence emission spectra of NIDCS-A in solution are shown in Figure 2a of the main text. UV-Vis absorption spectra were obtained using a Cary 50 Bio UV-Vis spectrometer over a range of 200-800 nm while the photoluminescence emission spectra were obtained using a homebuilt system. Photoluminescence quantum efficiency measurements were obtained in an integrating sphere using the method of de Mello et al.[4] The sample was illuminated by a laser diode (405 nm and 520 nm) collimated by an aspheric lens in a TE-cooled mount powered by a benchtop LD controller. Temperature was controlled by a benchtop temperature controller and the signal was detected by a Kymera 328i Andor Spectrograph with a detector (DU420A-BVF iDus). Results were calibrated against a known spectral source-Ocean optics, HL-3 plus, VIS-NIR light source.

2.2. Lack Of Photoswitching Under Alternating 450 and 520 nm Excitation ($PSS_{450} \approx PSS_{520}$)

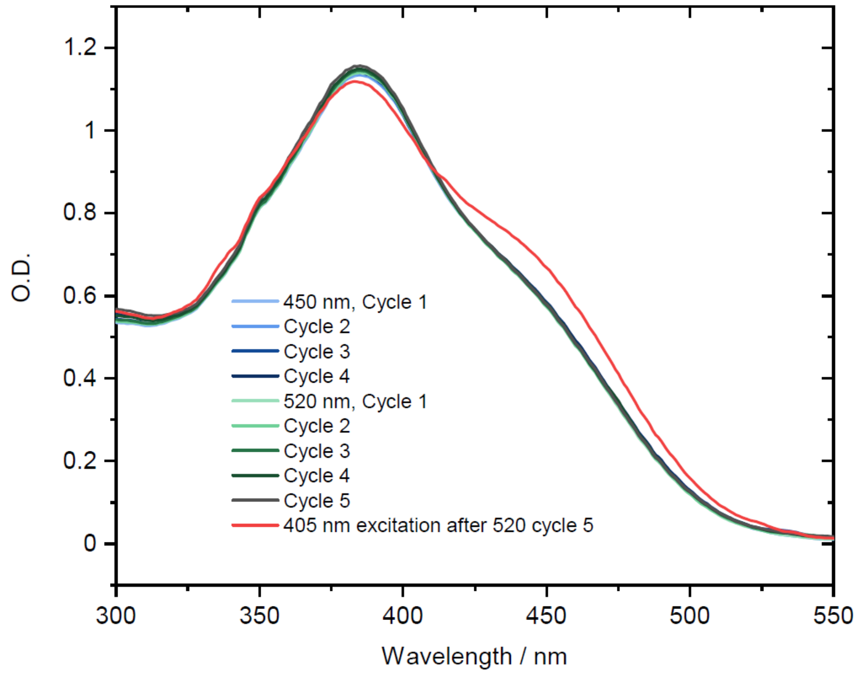

Figure S5. UV-Vis absorption spectra of NIDCS-A in $CHCl_3$ under alternating excitation at 450 and 520 nm.

2.3. Retention of NIDCS-A Photo-Switching in the Presence of Triplet Quenchers

2.3.1. O_2

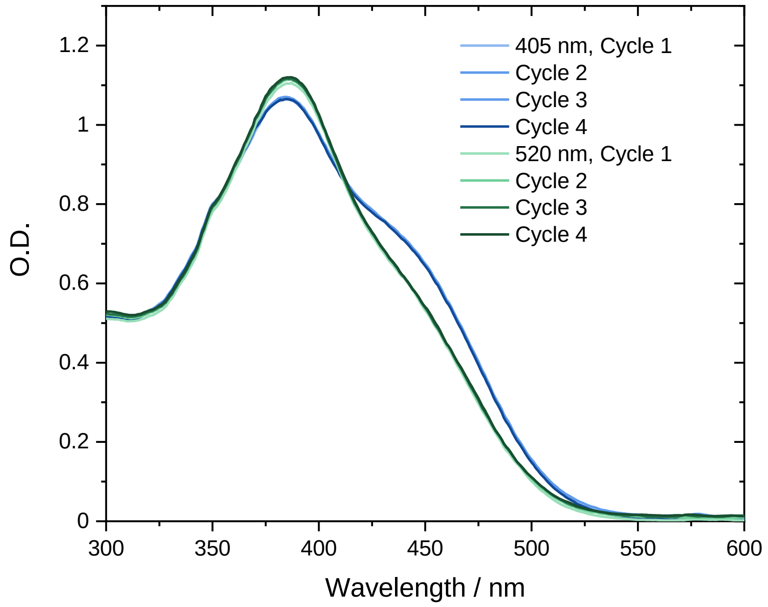

Figure S6. UV-Vis absorption spectra of NIDCS-A in $CHCl_3$ in an oxygen environment with alternating excitation at 405 and 520 nm.

2.3.2. Cyclooctatetraene (COT)

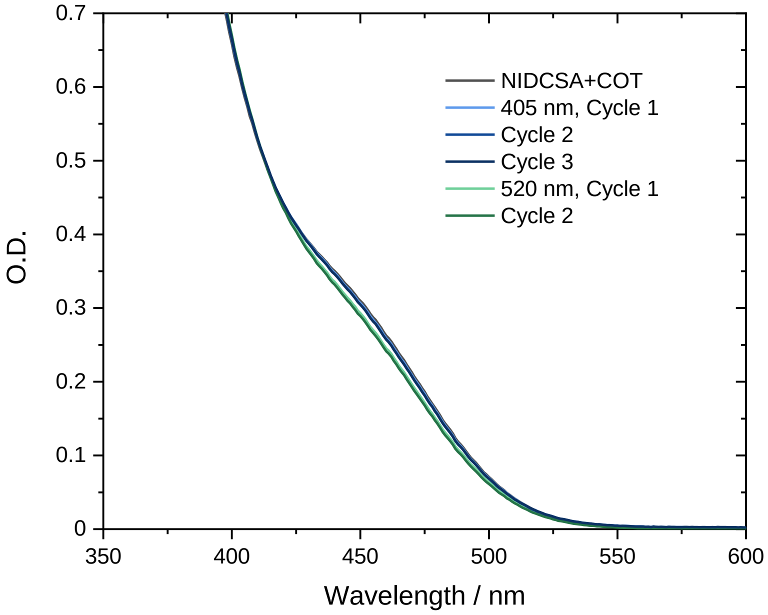

Figure S7. UV-Vis absorption spectra of NIDCS-A and COT in $CHCl_3$ under alternating excitation at 405 and 520 nm.

2.3.3. Photoswitching in Different Solvents

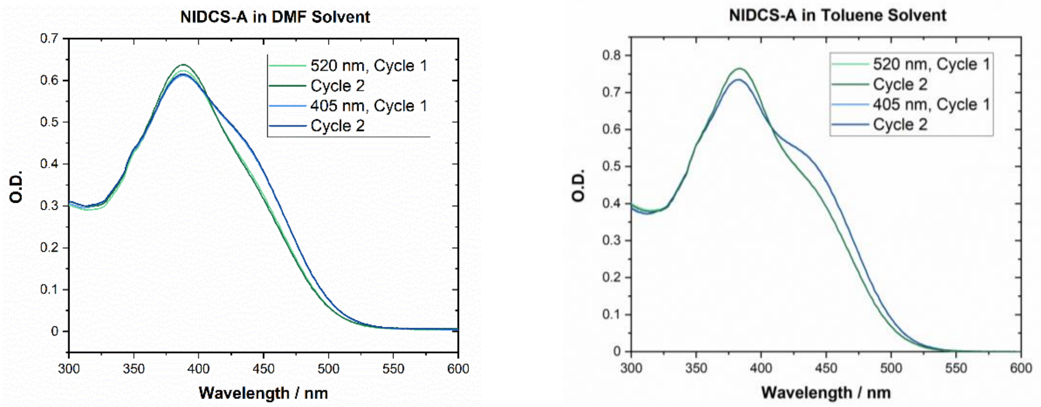

Figure S8. UV-Vis absorption spectra of NIDCS-A in DMF and toluene under alternating excitation at 405 and 520 nm.

2.4. Bounding of Isomerisation Timescale in the Triplet State

No suppression of NIDCS-A photoisomerization was observed in the presence of either oxygen or COT. In a saturated DCM solution, the concentration of O_2 is approximately 0.011 mol/L.[5] Combining this with a bimolecular quenching constant of ~10^{10} $M^{-1}s^{-1}$ yields a diffusion-limited triplet quenching rate of 1.1 × 10^8 s^{-1}. With COT, higher concentrations of quencher (0.3 M) could be obtained with NIDCS-A photoswitching retained. We employ the Stokes-Einstein equation ($D = {kT}/{6\pi\eta R}$ where k = Boltzmann's constant, T = temperature η = solvent viscosity, and R = molecular radius) to estimate the rates of diffusion of NIDCS-A ($R \approx 1$ nm) and COT ($R \approx 0.25$ nm) through solution as 4 × 10^{-6} and 1.3 ×10^{-5} cm^2s^{-1}, respectively. These values correspond to an estimated collision rate of 5 × 10^9 s^{-1} using the Smoluchowski equation and the concentration of COT (0.3 M).

2.5. Proton NMR Analysis of Photostationary States $PSS_{400\ nm}$ and $PSS_{520\ nm}$

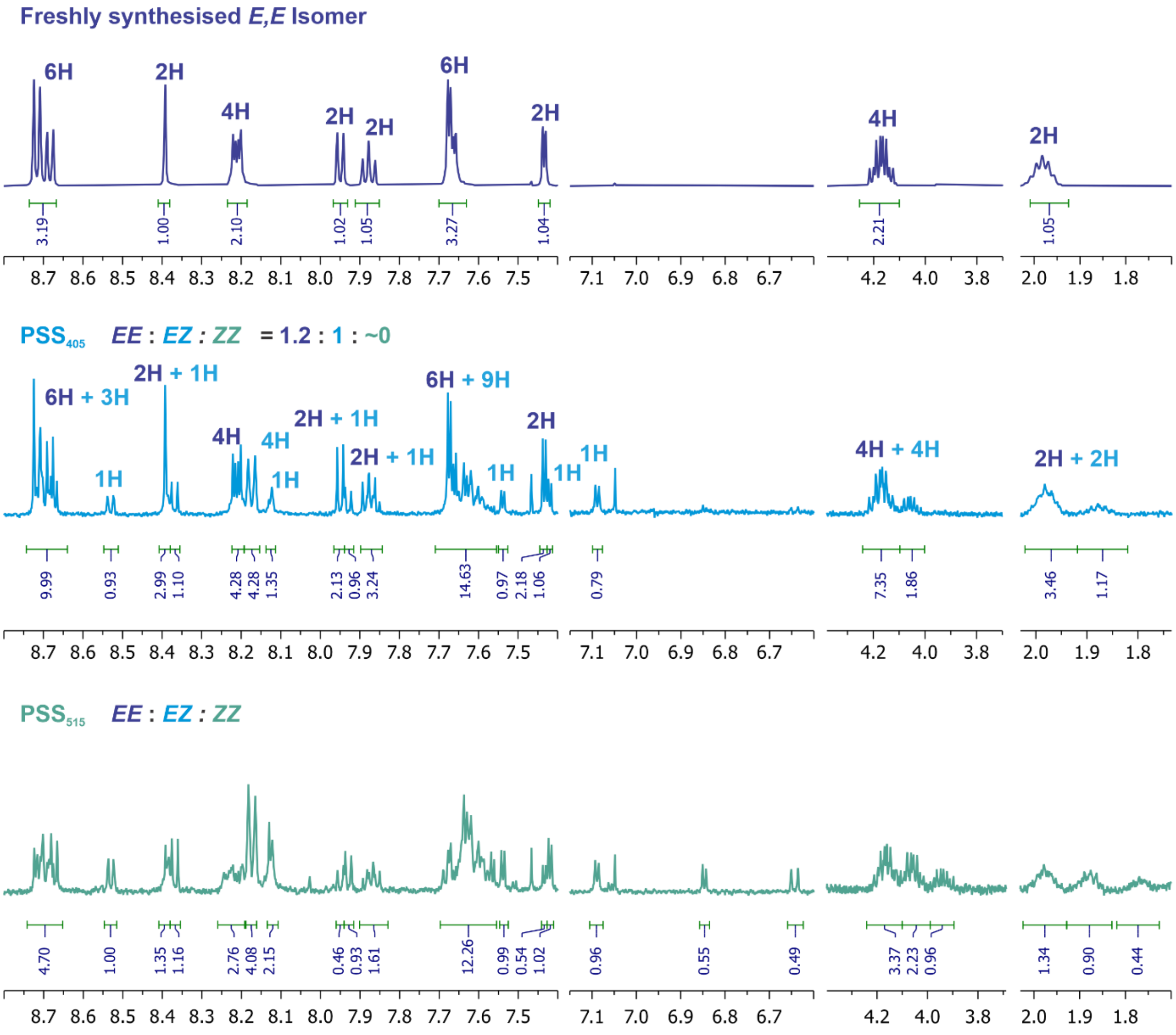

Figure S9. ^{1}H NMR spectra in $CDCl_3$ for freshly synthesised NIDCS-A, compared with PSS_{400} and PSS_{515}.

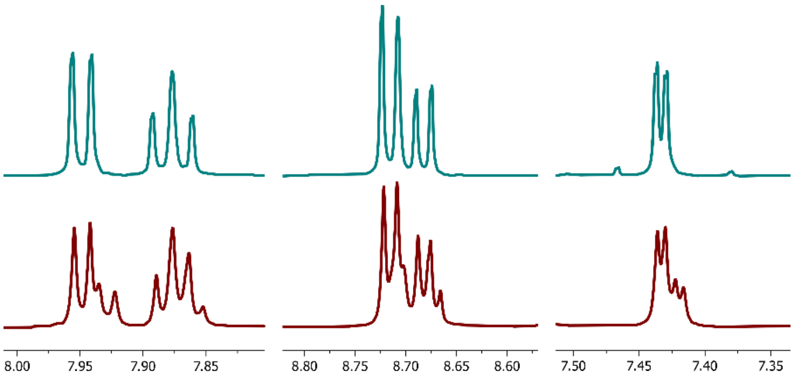

Figure S10. Zoomed in regions of ^{1}H NMR spectra of as-synthesised NIDCS-A (green) and PSS_{405} (maroon) in $CDCl_3$ showing the slight shifting of resonances from *E*-configuration substituent due to isomerisation of the other substituent.

2.6. 2D NMR Evidence for Isomerisation

In the freshly synthesised *E,E* material, the alkene proton resonance is readily identifiable as a singlet corresponding to 2 protons at 8.4 ppm. The presence of a single resonance indicates that both alkene protons are in equivalent nuclear environments due to molecular symmetry. The lack of splitting or correlations with other protons in the COSY spectrum confirms that this signal is from the alkene proton, because all other protons in the molecule have protons on neighboring carbon atoms. Importantly, the NOESY spectrum shows a weak through-space interactions with a multiplet around ~7.65 ppm, which is the signal of the protons on the 1, 4, 5, 8 positions of the anthracene core, i.e. those that are closest to the alkene.

The 2D spectra for $PSS_{400\ nm}$ are similar. The major isomer present appears to be the *EZ* configuration, as a new singlet develops at 8.15 ppm that is not coupled to other protons. The NOESY correlation between the alkene proton at 8.4 ppm with the anthracene resonance at 7.65 ppm remains. However, the newly developed alkene *H* resonance at 8.15 ppm lacks NOESY correlations to any other protons. This strongly suggests isomerisation, because the DFT-optimised geometries reveal that the distance in the *E* configuration is 2.37 Å, while in the *Z* configuration, this distance increases to 2.75 Å, consistent with the loss of observable NOESY interaction. Similar features can be observed in the 2D spectra of PSS_{520}.

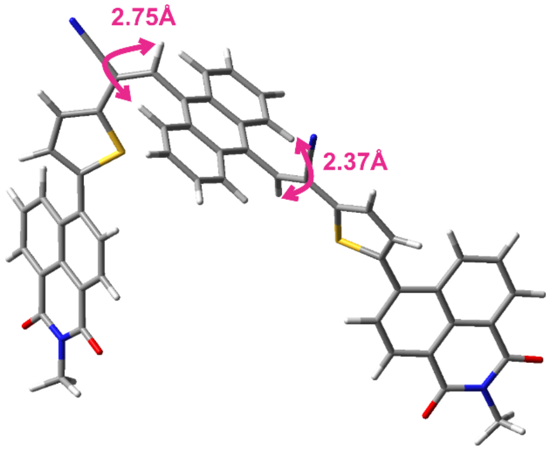

Figure S11. DFT-optimised geometry of *EZ* isomer showing changes in inter-proton distances consistent with the NOESY data.

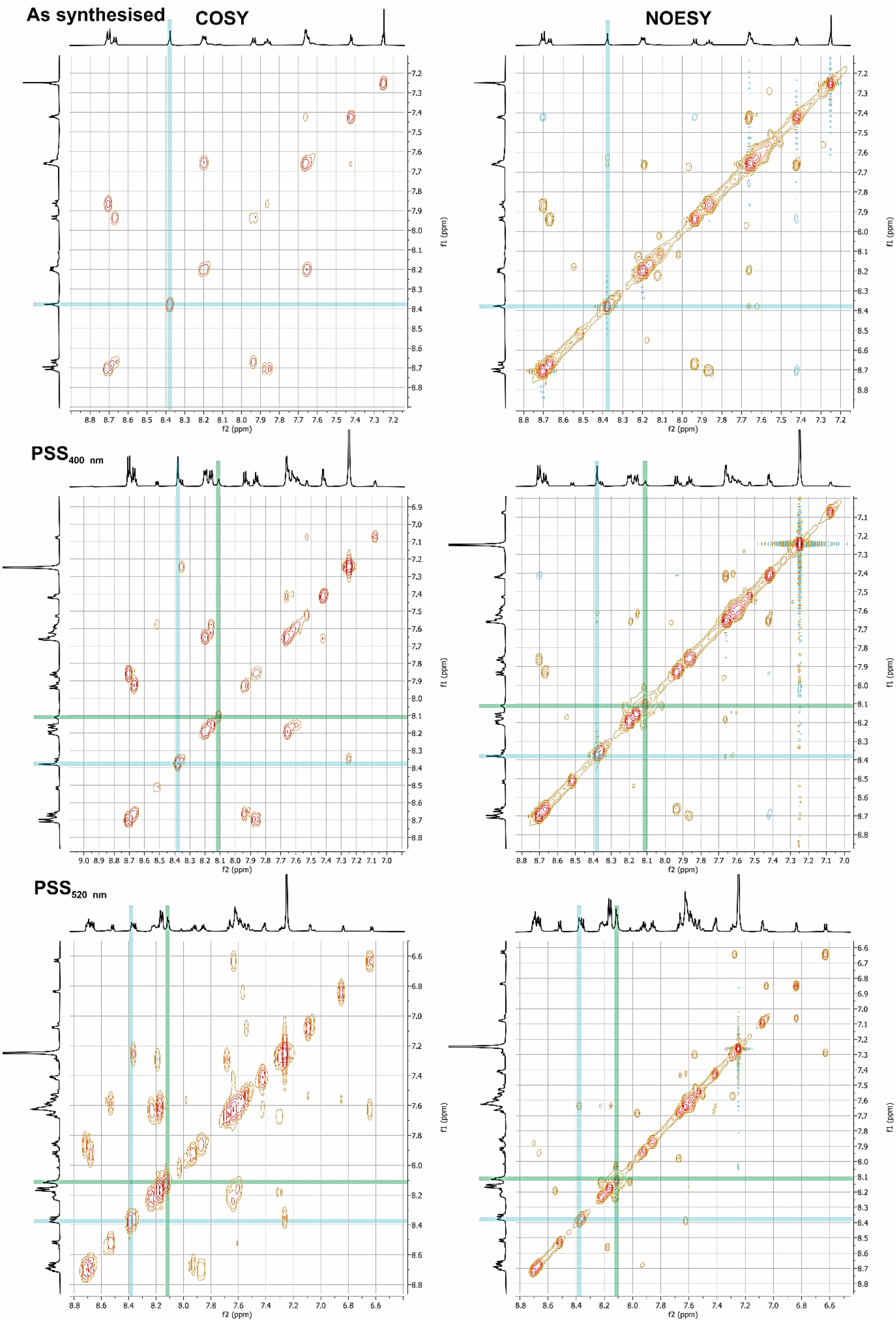

Figure S12. COSY and NOESY spectra of NIDCS-A, PSS_{405} and PSS_{515} in $CDCl_3$.

3. Time-Resolved Spectroscopy

3.1. Ultrafast Transient Absorption Spectroscopy

3.1.1. Experimental Details. Transient absorption measurements were carried out with either Ti-sapphire laser (Spectra Physics Solstice Ace), with pulse duration of 100 fs, centered at 800 nm, at a repetition rate of 1 kHz or femtosecond ytterbium fiber laser operating at 1-3 kHz with pulses <150 fs. Excitation/pump pulses were generated either using the fundamental, or by using an optical parametric amplifier (TOPAS) with the 800 nm fundamental input. Second harmonic excitation (400 nm) was generated directly from the fundamental using second harmonic generation crystal (BBO?), bypassing the TOPAS. Photoexcitations in the materials were probed via a broadband white light continuum generated by focusing a portion of the fundamental to an undoped Yttrium Aluminium Garnet (YAG) or sapphire crystal. After passing through the photoexcited sample, the probe pulses were spectrally dispersed using a prism spectrometer and collected using a CMOS camera (visible wavelengths) or an InGaAs photodiode array (IR wavelengths). Time resolution was obtained by introducing time delays in the pump path using a retroreflector mounted on a motorized translational stage. The pump-probe polarizations were set at the magic angle (54.7°) to avoid the effects of orientational dynamics.

3.1.2. Comparison of triplet formation under 400 nm and 515 nm excitation

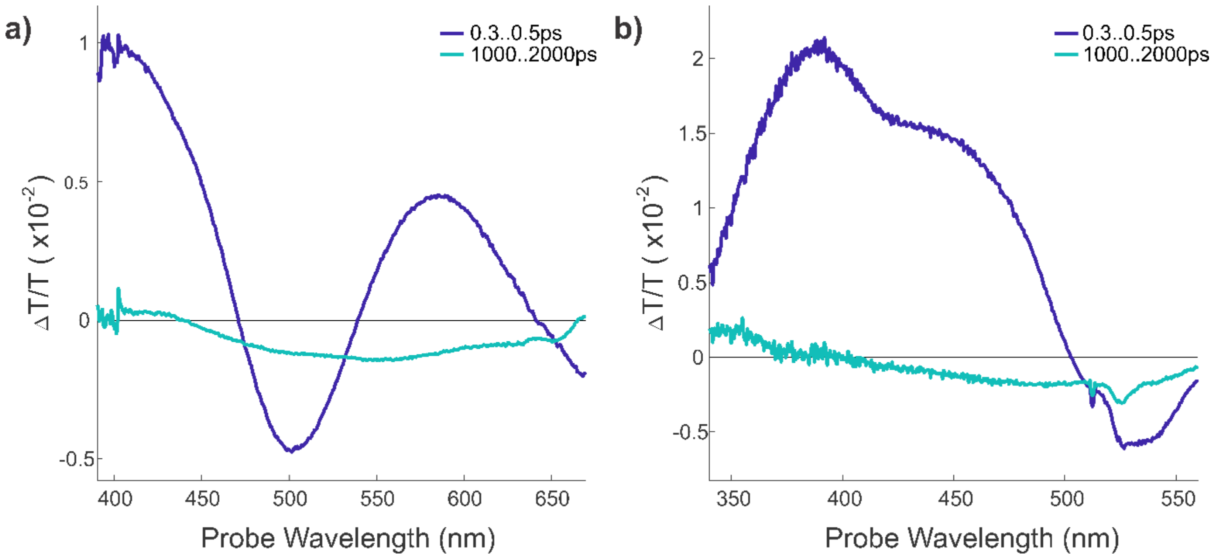

Figure S13. Transient absorption spectra of NIDCS-A solutions in dichloromethane (~10^{-4} M) showing similar triplet yields at nanosecond time delays under (a) 400 nm excitation and (b) 515 nm excitation.

3.1.3. Intensity Dependence of NIDCS-A Solution Transient Absorption Kinetics

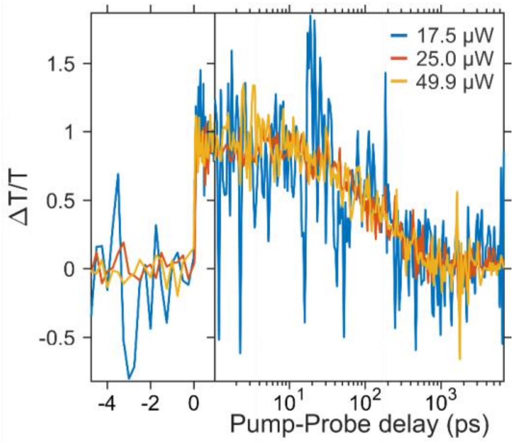

Figure S14. Ultrafast transient absorption kinetics of NIDCS-A solution in dichloromethane (~10^{-4} M) as a function of excitation power.

3.1.4. Ultrafast Transient Absorption Spectroscopy of NIDCS-A Thin Films

Thin films were prepared by spin-coating from 0.5 wt% chloroform solution at 1500 rpm for 60 s. Fused silica substrates used for preparing films were cleaned by sonicating in acetone followed by chloroform for 10 minutes each.

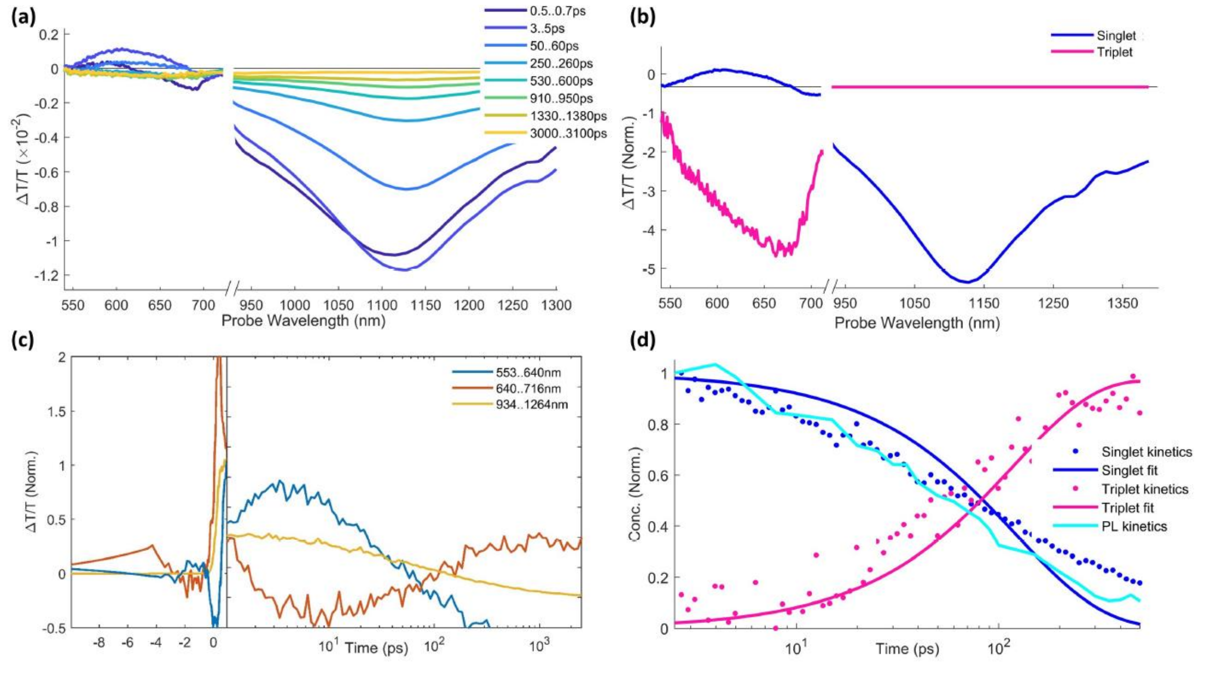

Figure S15. Ultrafast transient absorption spectra and kinetics of NIDCS-A film prepared by spin coating. (a) TA spectra (400 nm, 100 fs pump pulse), show the evolution from singlet (blue) to triplet (yellow). (b) TA spectra of the singlet (blue) and triplet (pink) excitons were obtained by applying a late time mask using the multivariate curve resolution alternating least squares (MCR-ALS) algorithm. [6] (c) Kinetics at different regions normalised at 5 ps. (d) TA kinetics extracted by applying MCR-ALS algorithm to the TA surface. Normalized kinetics of the extracted species fitted to the kinetic model (solid line) described in Reference [3]. Time-resolved PL kinetics matching the singlet kinetics are shown in cyan.

3.2. Ultrafast Transient Grating Photoluminescence Spectroscopy

3.2.1. Experimental Details. In this technique, the 1030 nm fundamental is split into two laser beams: the pump and gate beams. The pump beam is used to excite the sample.[7] The PL is collected and refocussed on to the gate medium (a 1 mm fused silica crystal) using a pair of off-axis parabolic mirrors. The gate beam is split in two using a 50/50 beam splitter and both beams were focussed on the gate medium at a crossing angle of approximately 8° and overlapping with the PL in a boxcar geometry. The two gate beams spatially and temporally overlap inside the gate medium, generating a laser-induced diffraction grating. This transient grating acts like an ultrafast optical shutter to temporally resolve the broadband PL signal by diffracting the gated signal away from the PL background.

3.3. Nanosecond-Microsecond Transient Absorption Spectroscopy

Experimental Details. The ns-TA measurements were performed on a commercial setup (Ultrafast Systems, Helios) pumped by a titanium:sapphire (Ti:Saph) amplifier (Spectra Physics Solstice Ace) that generates an 800 nm pulse train (1 kHz, 100 fs, Figure 3.4). The 800nm output was focused on a YAG or sapphire or CaF_2 crystal to generate the white light continuum probe depending on the measurement range. The CaF_2 crystal is kept in constant motion with a motorized stage. A ND:YVO_4 laser system (Piccolo Innolas, 355nm or 532 nm, 800 ps pulse duration) set to external frequency (500 Hz) control was used as the pump beam. A grid polariser and a half-wave plate set the pump beam to the magic angle (54.7°) relative to the probe. The pump-probe delay was controlled electronically via a delay generator, triggering the pump pulse. The signal was detected with a silicon linear array detector for the visible range or a InGaAs linear array detector for the infrared range.

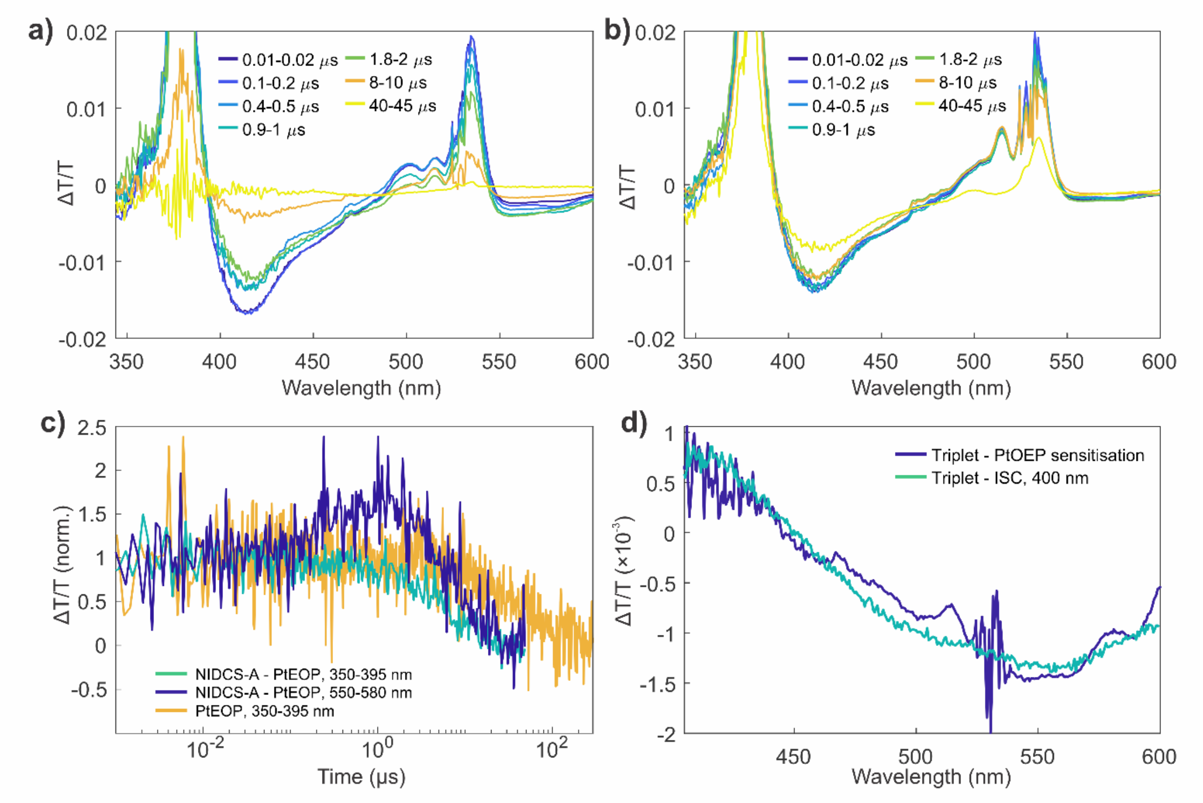

Figure S16. Nanosecond-microsecond transient absorption spectroscopy of photosensitisation of NIDCS-A triplets via excitation of platinum octaethylporphyrin (PtOEP). (a) Spectral slices from ns-μs TA spectrum of solution of NIDCS-A (2 ×10^{-5} M) and PtOEP in dichloromethane, pumped at 532 nm to excite PtOEP. (b) Spectral slices from ns-μs TA spectrum of solution of PtOEP in dichloromethane, pumped at 532 nm. (c) Kinetics from ns-μs TA spectra in parts a) and b) showing the rise of the PIA at 550-580 nm, and the accelerated decay of the PtOEP triplet bleach. (d) NIDCS-A triplet spectrum obtained from sensitisation data by subtracting the scaled PtOEP TA spectrum (10-50 ns) from the TA spectrum at 0.5-1.0 μs (blue). NIDCS-A triplet spectrum (5-6 ns; pink) observed for NIDCS-A in dichloromethane following excitation at 400 nm (c.f. Figure 3a in the main text).

3.4. Calculation of Triplet Yield

The triplet yield due to ISC in NIDCS-A was determined by using the sensitisation experiment with PtOEP to calculate the absorption cross-section of NIDCS-A triplets at 550 nm, and comparing this to the triplet signal from ISC in pure NIDCS-A.

3.4.1. NIDCS-A Triplet Cross-section

In the sensitisation measurement, the excitation density of PtOEP excited states generated by the pump c_{PtOEP} is:

$$c_{PtOEP} = I(1 - 10^{-OD_{532}}) = 2.61 \times 10^{13}\ \mathrm{cm}^{-2}$$

Where $I = 9.37 \times 10^{13}\ \mathrm{cm}^{-2}$ is the photon density per pulse (corresponding to a fluence of 35 μJ/cm^2 at 532 nm), and $OD_{532} = 0.142$ is the optical density of the PtOEP-NIDCS-A solution at the pump wavelength.

The NIDCS-A triplet signature reaches peak amplitude at a time delay of ~1 μs. By this time, the GSB of PtOEP centred at 380 nm decreases by 15%. This decay can be attributed purely to collisional quenching between PtOEP and NIDCS-A, because the pure PtOEP signal does not decay appreciably on this timescale (c.f. Figure S16). This corresponds to a density of NIDCS-A triplets of $c_{NIDCS-A} = 3.92 \times 10^{12}\ \mathrm{cm}^{-2}$.

The peak amplitude of the NIDCS-A triplet PIA at 550 nm in the sensitisation experiment is $\Delta T/T = -0.0015$ (c.f. Figure S16d). This can be combined with the triplet concentration to calculate the NIDCS-A triplet absorption cross-section at 550 nm:

$$\sigma_{NIDCS-A} = \frac{\Delta A}{c_{NIDCS-A}} = \frac{-log_{10}\left(\frac{\Delta T}{T} + 1\right)}{c_{NIDCS-A}} = 1.66 \times 10^{-16}\ cm^2$$

3.4.2. NIDCS-A Triplet Yield

In the TA measurement of pure NIDCS-A the excitation density of NIDCS-A singlet excited states initially generated by the pump c_0 is:

$$c_0 = I(1 - 10^{-OD_{400}}) = 1.45 \times 10^{13}\ \mathrm{cm}^{-2}$$

Where $I = 2.27 \times 10^{13}\ \mathrm{cm}^{-2}$ is the photon density per pulse (corresponding to a fluence of 11.3 μJ/cm^2 at 400 nm), and $OD_{400} = 0.439$ is the optical density of NIDCS-A at the pump wavelength.

The number of triplets eventually formed by ISC is calculated from the residual PIA at 550 nm, which has $\Delta T/T = -0.0015$ (coincidentally the same as in the experiment with PtOEP).

$$c_{NIDCS-A} = \frac{\Delta A}{\sigma_{NIDCS-A}} = \frac{-log_{10}\left(\frac{\Delta T}{T} + 1\right)}{\sigma_{NIDCS-A}} = 3.92 \times 10^{12}\ cm^{-2}$$

The ISC yield φ_{ISC} is then:

$$\varphi_{ISC} = \frac{c_{NIDCS-A}}{c_0} = 0.27 = 27\%$$

4. Theoretical calculations

4.1. Methods

Density functional theory (DFT) calculations were performed in Gaussian 16[8] using the CAM-B3LYP[9] exchange–correlation functional in combination with the 6-31G(d,p) basis set. Linear response time dependent DFT (TD-DFT) calculations for excited states were performed within the Tamm-Dankoff approximation (TDA). Solvent effects were included using a polarizable continuum model (integral equation formalism) with dielectric parameters for dichloromethane. Transition states were verified via subsequent intrinsic reaction coordinate calculations.

4.2. Photoisomerisation Potential Energy Surface

Figure S17 shows the DFT-calculated energy of NIDCS-A along the intrinsic reaction coordinate (IRC) associated with photoisomerisation.

The top plot shows the IRC in the excited state proceeding downhill from the transition state at step ~90. An avoided crossing or conical intersection develops at step 101 soon after the transition state in S_1. It should be noted that conical intersections cannot be calculated using TD-DFT, but the surrounding regions can be, including as the transition state, which still has a S_1-S_0 gap of ~1 eV.

The second plot was constructed by perturbing the molecular geometry along the imaginary frequency associated with the transition state, to avoid the surface crossing region where TD-DFT becomes invalid (and where the previous IRC calculation terminates). This corresponds to a change in the H-C=C-CN dihedral angle from 82.0° at step 102 of the top plot, to -76.7° in step 1 the bottom plot. The resulting geometry was then used as the starting point for an IRC calculation proceeding downhill in the electronic ground state (to reflect the likely $S_1 \rightarrow S_0$ internal conversion during the photoisomerisation process). Excited state energies were calculated as single point TD-DFT energies.

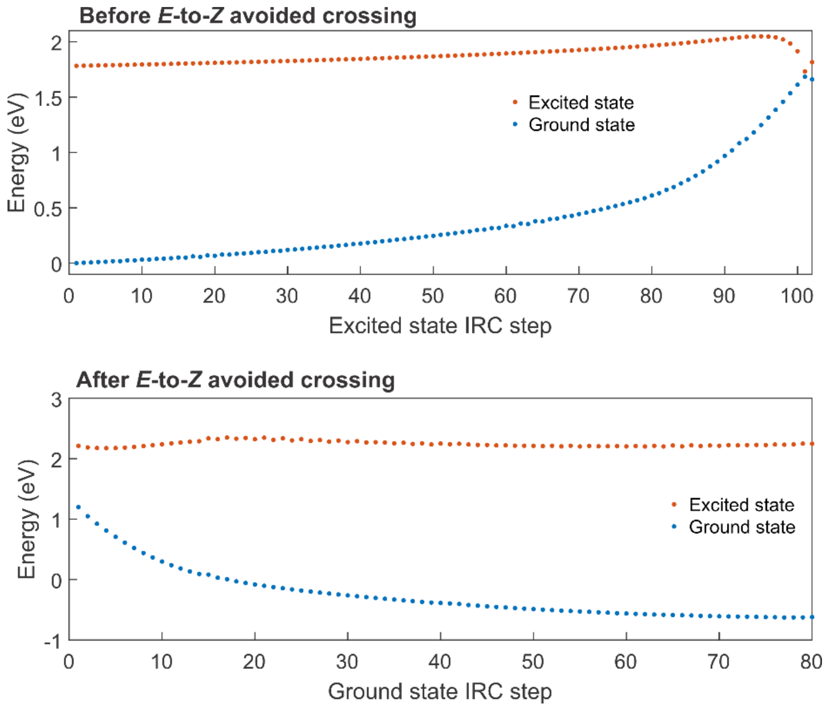

Figure S17. Ground and excited state energies of NIDCS-A as a function of progression along the intrinsic reaction coordinate for photoisomerisation.

4.3. Optimised Geometries and State Energies

***(E,E)*-S_0**

$E(S_0)$ (Ha) = −3390.84429450Ha

$E(S_1)$ −$E(S_0)$ = 3.13 eV

$E(T_1)$ − $E(S_0)$ = 2.00 eV

C	-0.82095000	1.00366400	0.55918400
C	0.60349000	1.07093000	0.71251000
C	1.40669000	0.05652100	0.15711900
C	2.88065800	0.09896600	0.26353700
H	3.42795200	0.20909000	-0.67084600
C	3.60264200	-0.02104600	1.39318400
C	2.93310100	-0.24684200	2.64898000
N	2.43005800	-0.43864200	3.67242500
C	5.06546600	0.02753700	1.47320100
C	5.83518900	-0.14642100	2.59123600
S	6.05942100	0.31334700	0.07836900
C	7.22725900	-0.05572000	2.33239500
H	5.41663700	-0.32993500	3.57296100
C	7.51888700	0.19096800	1.01899700
H	7.98499700	-0.13933900	3.10089900
C	8.82810500	0.40605600	0.38040500
C	9.92479800	-0.49126200	0.60021100
C	9.00566200	1.50071300	-0.44387400
C	9.82259500	-1.68461700	1.35914300
C	11.17655700	-0.19054700	0.00071300
C	10.24320800	1.77441100	-1.04393900
H	8.17942600	2.18336500	-0.60697400
C	10.90695900	-2.50566600	1.54409500
H	8.86645300	-1.95760300	1.78652400
C	12.28188100	-1.04784200	0.21110100
C	11.32036200	0.95278700	-0.81808800
H	10.36919800	2.64418700	-1.67759900
C	12.15072900	-2.18203700	0.97601600
H	10.80296400	-3.41487700	2.12517700
C	13.59230800	-0.73710200	-0.39986600
C	12.62026000	1.27794600	-1.44573300
H	13.01464700	-2.82071700	1.11758500
O	14.57856100	-1.43770000	-0.23829000
N	13.67801000	0.40676000	-1.18870600
O	12.77080600	2.25914800	-2.15570700
C	14.98294000	0.68809000	-1.78254800
H	15.72841000	0.81462100	-0.99674100
H	15.28728600	-0.14328700	-2.41918300
H	14.89349200	1.59793600	-2.36760000
C	-1.61815500	2.05022500	1.12180400
H	-2.69401100	2.01512800	0.99625200
C	1.15632200	2.20080800	1.39553200
H	2.23197000	2.28596300	1.48136200
C	0.36065600	3.17187800	1.91987300
H	0.80494100	4.01900800	2.43116900
C	-1.05169300	3.09172100	1.78906900
H	-1.67565000	3.87337900	2.20873800
C	0.82095000	-1.00366400	-0.55918400
C	-0.60349000	-1.07093000	-0.71251000
C	-1.40669000	-0.05652100	-0.15711900
C	-2.88065800	-0.09896600	-0.26353700
H	-3.42795200	-0.20909000	0.67084600
C	-3.60264200	0.02104600	-1.39318400
C	-2.93310100	0.24684200	-2.64898000
N	-2.43005800	0.43864200	-3.67242500
C	-5.06546600	-0.02753700	-1.47320100
C	-5.83518900	0.14642100	-2.59123600
S	-6.05942100	-0.31334700	-0.07836900
C	-7.22725900	0.05572000	-2.33239500
H	-5.41663700	0.32993500	-3.57296100
C	-7.51888700	-0.19096800	-1.01899700
H	-7.98499700	0.13933900	-3.10089900
C	-8.82810500	-0.40605600	-0.38040500
C	-9.92479800	0.49126200	-0.60021100
C	-9.00566200	-1.50071300	0.44387400
C	-9.82259500	1.68461700	-1.35914300
C	-11.17655700	0.19054700	-0.00071300
C	-10.24320800	-1.77441100	1.04393900
H	-8.17942600	-2.18336500	0.60697400
C	-10.90695900	2.50566600	-1.54409500
H	-8.86645300	1.95760300	-1.78652400
C	-12.28188100	1.04784200	-0.21110100
C	-11.32036200	-0.95278700	0.81808800
H	-10.36919800	-2.64418700	1.67759900
C	-12.15072900	2.18203700	-0.97601600
H	-10.80296400	3.41487700	-2.12517700
C	-13.59230800	0.73710200	0.39986600
C	-12.62026000	-1.27794600	1.44573300
H	-13.01464700	2.82071700	-1.11758500
O	-14.57856100	1.43770000	0.23829000
N	-13.67801000	-0.40676000	1.18870600
O	-12.77080600	-2.25914800	2.15570700
C	-14.98294000	-0.68809000	1.78254800
H	-15.72841000	-0.81462100	0.99674100
H	-15.28728600	0.14328700	2.41918300
H	-14.89349200	-1.59793600	2.36760000
C	1.61815500	-2.05022500	-1.12180400
H	2.69401100	-2.01512800	-0.99625200
C	-1.15632200	-2.20080800	-1.39553200
H	-2.23197000	-2.28596300	-1.48136200
C	-0.36065600	-3.17187800	-1.91987300
H	-0.80494100	-4.01900800	-2.43116900
C	1.05169300	-3.09172100	-1.78906900

H	1.67565000	-3.87337900	-2.20873800

(*E*,*E*)-S_1

$E(S_0)$ (Ha) = −3390.82509360 Ha

$E(S_1) - E(S_0)$ = 2.03 eV

$E(T_1) - E(S_0)$ = 0.99 eV

C	-0.84441400	0.84915800	0.79009300
C	0.57215500	0.89423600	0.96056800
C	1.43449700	0.01071700	0.21319100
C	2.83551100	-0.05825400	0.42119000
H	3.42785200	-0.28208500	-0.46032700
C	3.58145900	0.01842000	1.59145100
C	2.96969500	-0.09404500	2.87855200
N	2.51992100	-0.21641700	3.94017100
C	5.01840000	0.04522700	1.60997200
C	5.85360000	-0.06064600	2.71061200
S	5.96211300	0.22166000	0.15362900
C	7.21872600	-0.01843300	2.38289500
H	5.47799600	-0.16034700	3.72102100
C	7.46263000	0.12988300	1.03400400
H	8.00829900	-0.05348500	3.12219400
C	8.73823100	0.29117800	0.33702300
C	9.89348600	-0.49385200	0.67735200
C	8.84454000	1.23879300	-0.66955500
C	9.86931900	-1.56818400	1.60080300
C	11.12249500	-0.21208000	0.02232200
C	10.05690700	1.48535200	-1.32257600
H	7.97963400	1.83883400	-0.92853600
C	11.00622100	-2.27825300	1.90103400
H	8.93160300	-1.84945200	2.06149900
C	12.28274400	-0.95038400	0.35394900
C	11.19000000	0.78793600	-0.97437600
H	10.12396400	2.24431200	-2.09290500
C	12.22746400	-1.95934400	1.28614900
H	10.95895100	-3.09830900	2.60843300
C	13.57029000	-0.65282000	-0.30961800
C	12.46294400	1.09284400	-1.66008600
H	13.13243400	-2.50867300	1.51764500
O	14.60261100	-1.24799800	-0.04402400
N	13.57854400	0.34977700	-1.27460100
O	12.54696700	1.95178300	-2.52380600
C	14.86183800	0.61957200	-1.91843500
H	15.59333900	0.93127300	-1.17202000
H	15.22820900	-0.28376000	-2.40720200
H	14.70878300	1.40819300	-2.64839400
C	-1.63140300	1.75646600	1.53274500
H	-2.70571400	1.76598700	1.41033000
C	1.11213800	1.91411600	1.77374400
H	2.18378500	2.05097200	1.80420100
C	0.31542200	2.77452300	2.49700000
H	0.76911800	3.53855000	3.11810200
C	-1.07380400	2.67656600	2.39457900
H	-1.71509800	3.35027200	2.95180700
C	0.84441400	-0.84915800	-0.79009300
C	-0.57215500	-0.89423600	-0.96056800
C	-1.43449700	-0.01071700	-0.21319100
C	-2.83551100	0.05825400	-0.42119000
H	-3.42785200	0.28208500	0.46032700
C	-3.58145900	-0.01842000	-1.59145100
C	-2.96969500	0.09404500	-2.87855200
N	-2.51992100	0.21641700	-3.94017100
C	-5.01840000	-0.04522700	-1.60997200
C	-5.85360000	0.06064600	-2.71061200
S	-5.96211300	-0.22166000	-0.15362900
C	-7.21872600	0.01843300	-2.38289500
H	-5.47799600	0.16034700	-3.72102100
C	-7.46263000	-0.12988300	-1.03400400
H	-8.00829900	0.05348500	-3.12219400
C	-8.73823100	-0.29117800	-0.33702300
C	-9.89348600	0.49385200	-0.67735200
C	-8.84454000	-1.23879300	0.66955500
C	-9.86931900	1.56818400	-1.60080300
C	-11.12249500	0.21208000	-0.02232200
C	-10.05690700	-1.48535200	1.32257600
H	-7.97963400	-1.83883400	0.92853600
C	-11.00622100	2.27825300	-1.90103400
H	-8.93160300	1.84945200	-2.06149900
C	-12.28274400	0.95038400	-0.35394900
C	-11.19000000	-0.78793600	0.97437600
H	-10.12396400	-2.24431200	2.09290500
C	-12.22746400	1.95934400	-1.28614900
H	-10.95895100	3.09830900	-2.60843300
C	-13.57029000	0.65282000	0.30961800
C	-12.46294400	-1.09284400	1.66008600
H	-13.13243400	2.50867300	-1.51764500
O	-14.60261100	1.24799800	0.04402400
N	-13.57854400	-0.34977700	1.27460100
O	-12.54696700	-1.95178300	2.52380600
C	-14.86183800	-0.61957200	1.91843500
H	-15.59333900	-0.93127300	1.17202000
H	-15.22820900	0.28376000	2.40720200
H	-14.70878300	-1.40819300	2.64839400
C	1.63140300	-1.75646600	-1.53274500
H	2.70571400	-1.76598700	-1.41033000
C	-1.11213800	-1.91411600	-1.77374400
H	-2.18378500	-2.05097200	-1.80420100
C	-0.31542200	-2.77452300	-2.49700000
H	-0.76911800	-3.53855000	-3.11810200
C	1.07380400	-2.67656600	-2.39457900
H	1.71509800	-3.35027200	-2.95180700

(*E*,*E*) -S_1 →(*E*,*Z*)-S_1 TS

$E(S_0)$ (Ha) = -3390.76833227 Ha

$E(S_1) - E(S_0)$ = 1.01 eV

$E(T_1) - E(S_0)$ = −0.33 eV

C	0.40024200	-0.73077900	-0.34186400

C	-0.98405600	-1.06822100	-0.34944200
C	-1.64748100	-1.39718100	0.91193900
C	-2.77040200	-2.17142000	1.00603900
H	-3.09895700	-2.41192400	2.01627000
C	-3.55402200	-2.82022000	-0.06071600
C	-3.02201500	-3.97715900	-0.65997500
N	-2.56997400	-4.93037700	-1.15775300
C	-4.84475200	-2.39807000	-0.38489000
C	-5.76265600	-2.92951400	-1.31116000
S	-5.54583700	-0.99636500	0.40486300
C	-6.96567700	-2.23550600	-1.35437700
H	-5.52821000	-3.78482600	-1.93176900
C	-7.05510300	-1.14679000	-0.49100600
H	-7.75465700	-2.48499500	-2.05248900
C	-8.09347400	-0.15920600	-0.35065000
C	-9.49321100	-0.46679000	-0.54870800
C	-7.75569500	1.15796900	-0.02193200
C	-9.99037100	-1.78236100	-0.69182000
C	-10.43949500	0.59593400	-0.53900400
C	-8.70230000	2.17530800	0.01899500
H	-6.71423800	1.41085800	0.14086800
C	-11.32732100	-2.03131400	-0.90589700
H	-9.30926500	-2.61710300	-0.59749000
C	-11.80727300	0.32036600	-0.77486100
C	-10.03088200	1.92078200	-0.26464600
H	-8.40459500	3.19334200	0.24223800
C	-12.24111300	-0.97170000	-0.97105600
H	-11.67671000	-3.05265100	-1.00645200
C	-12.79403700	1.41901000	-0.79390600
C	-10.99490900	3.02752000	-0.25935600
H	-13.29622300	-1.14786100	-1.14484500
O	-13.98257300	1.23813200	-1.01889300
N	-12.32669500	2.70285400	-0.53963000
O	-10.67232900	4.18492300	-0.02555200
C	-13.32181300	3.77068600	-0.55928400
H	-13.79380700	3.82164400	-1.54135800
H	-14.09425800	3.57358200	0.18512600
H	-12.81259500	4.70303400	-0.33649300
C	1.04403500	-0.44500200	-1.57445100
H	2.08880600	-0.16155900	-1.57814100
C	-1.66617900	-1.05865700	-1.57244700
H	-2.72823500	-1.25304200	-1.58830600
C	-1.01060200	-0.78000100	-2.75678300
H	-1.56872900	-0.77615600	-3.68671600
C	0.35801300	-0.48326600	-2.76196200
H	0.86618600	-0.25930100	-3.69274400
C	-0.96403500	-1.01403800	2.15274800
C	0.43273000	-0.72428500	2.13294000
C	1.10895700	-0.59218400	0.88802200
C	2.53659800	-0.30077000	0.81534400
H	3.11025200	-0.96820900	0.17694600
C	3.20986400	0.73659100	1.37179900
C	2.50456500	1.78556000	2.06173400
N	1.97450800	2.66062200	2.60056400
C	4.64498700	0.95063300	1.24929300
C	5.35303600	2.04756100	1.67542600
S	5.69965800	-0.21058300	0.50189500
C	6.73692600	1.96057400	1.39750200
H	4.89204300	2.88864800	2.17794800
C	7.09052800	0.79749100	0.76428500
H	7.45380600	2.71877600	1.68474600
C	8.42761800	0.32494900	0.37019900
C	9.33423800	1.16559300	-0.35591000
C	8.81901200	-0.95411000	0.71644700
C	8.99871800	2.45914500	-0.82989200
C	10.63099700	0.66639400	-0.64844600
C	10.09765600	-1.43872600	0.40506500
H	8.13667000	-1.58858900	1.27070000
C	9.90953500	3.22393700	-1.51501300
H	8.00077100	2.84227500	-0.66031100
C	11.55495000	1.47534500	-1.35036500
C	10.99923500	-0.64064200	-0.25521400
H	10.39447300	-2.43941700	0.69533200
C	11.20240400	2.73565300	-1.76952600
H	9.62881900	4.20877500	-1.87050600
C	12.91178500	0.96824200	-1.65001100
C	12.34952900	-1.17043000	-0.55155000
H	11.93063000	3.33203000	-2.30648600
O	13.74806600	1.63187600	-2.24096700
N	13.22232600	-0.32260700	-1.23117300
O	12.69151600	-2.29406400	-0.22080000
C	14.56778900	-0.79934500	-1.54342700
H	15.30902500	-0.15453000	-1.07012300
H	14.72685500	-0.77813400	-2.62194100
H	14.65684300	-1.81410000	-1.16870500
C	-1.63306300	-0.97387300	3.38323900
H	-2.70878400	-1.08992500	3.41637700
C	1.11863200	-0.59810400	3.37187100
H	2.19532800	-0.50072600	3.37439800
C	0.44299100	-0.61392100	4.56381800
H	0.98214500	-0.50675100	5.49776000
C	-0.95102600	-0.76454200	4.56502000
H	-1.49876500	-0.73505100	5.50060400

(E,Z)-S_0

$E(S_0)$ (Ha) = −3390.84310811 Ha

$E(S_1)$ −$E(S_0)$ = 3.26 eV

$E(T_1)$ − $E(S_0)$ =2.06 eV

C	-1.27860600	-3.11545100	1.26635500
C	-2.63280000	-3.55895800	1.40554300
C	-3.38610900	-3.86286300	0.25820000
C	-4.76464500	-4.39014800	0.41214000
H	-4.85376700	-5.46084000	0.58246200
C	-5.90957300	-3.68296400	0.36880000
C	-7.13165900	-4.43168300	0.54073000

N	-8.11425300	-5.02600200	0.67715300
C	-6.10374600	-2.24282600	0.17278100
C	-7.31701200	-1.60980900	0.08300000
S	-4.80602600	-1.09390600	0.04778900
C	-7.21080900	-0.20820000	-0.08750800
H	-8.26123800	-2.13748800	0.13254300
C	-5.91502800	0.23134200	-0.12808400
H	-8.06461700	0.44610200	-0.20821600
C	-5.40336100	1.59416200	-0.34988800
C	-5.90885500	2.71438500	0.38886000
C	-4.41784100	1.79885500	-1.29661200
C	-6.85687500	2.59948200	1.43694500
C	-5.41337500	4.00892400	0.08031600
C	-3.92454400	3.08130900	-1.57678400
H	-4.03841600	0.95417400	-1.86032100
C	-7.31414100	3.70642000	2.10764600
H	-7.21292700	1.61749000	1.72037500
C	-5.90493300	5.13596500	0.77969000
C	-4.42045500	4.17611600	-0.91199000
H	-3.16068100	3.22593600	-2.33144600
C	-6.84487200	4.98744700	1.77132900
H	-8.03631200	3.59280300	2.90809000
C	-5.40471900	6.48948100	0.45622500
C	-3.89870500	5.52007200	-1.24428400
H	-7.20131800	5.86890900	2.29122200
O	-5.80693300	7.49417000	1.02064700
N	-4.43426500	6.59525200	-0.53635400
O	-3.04105600	5.69141000	-2.09562200
C	-3.95554900	7.94319300	-0.83395000
H	-4.78792600	8.56871800	-1.15780900
H	-3.51532400	8.38650000	0.05988700
H	-3.21254300	7.86640400	-1.62146000
C	-0.53259700	-2.83160400	2.45313900
H	0.50157700	-2.52027900	2.36245400
C	-3.18086100	-3.68314800	2.72066200
H	-4.20733900	-4.01427200	2.82815300
C	-2.43899900	-3.39217900	3.82395400
H	-2.87215200	-3.49346100	4.81317800
C	-1.09232100	-2.95953700	3.68755700
H	-0.50850700	-2.73707000	4.57422000
C	-2.81911200	-3.74351400	-1.02135100
C	-1.46649500	-3.28257200	-1.16430100
C	-0.70795000	-2.99006400	-0.01495500
C	0.68236400	-2.49763400	-0.11382300
H	0.86392600	-1.49114600	0.25840400
C	1.74167900	-3.18550300	-0.57929600
C	1.57559800	-4.55103000	-1.00813000
N	1.48231200	-5.65168600	-1.35026900
C	3.11211800	-2.67319100	-0.66834500
C	4.22484900	-3.36333500	-1.06633600
S	3.51942700	-1.04006800	-0.24125200
C	5.41089900	-2.58577000	-1.02446300
H	4.19505800	-4.39812800	-1.38406200
C	5.20236500	-1.30258700	-0.59927100
H	6.38067900	-2.95765400	-1.32949100
C	6.16351400	-0.19359500	-0.47887000
C	7.41683000	-0.35956800	0.19766600
C	5.85308300	1.03248000	-1.03510200
C	7.79354800	-1.54908800	0.87119700
C	8.32132900	0.73481700	0.22589200
C	6.74971600	2.10954200	-0.98397900
H	4.90819300	1.15851100	-1.55145000
C	9.00965800	-1.65707200	1.49851400
H	7.10052300	-2.37984900	0.90169600
C	9.57176000	0.59807800	0.87298700
C	7.97282500	1.96551900	-0.37604000
H	6.49624300	3.06069600	-1.43669000
C	9.91272200	-0.58054000	1.49215900
H	9.27377000	-2.57550000	2.01024700
C	10.52446400	1.72892600	0.89879600
C	8.90827600	3.11185100	-0.35597300
H	10.87673000	-0.65532700	1.98146500
O	11.61884400	1.65774800	1.43441100
N	10.13271900	2.91309200	0.28046700
O	8.63183100	4.18419200	-0.86888700
C	11.09187600	4.01443800	0.32280400
H	12.02283400	3.71339000	-0.15861800
H	11.30673600	4.27977200	1.35854200
H	10.65267900	4.85878200	-0.19901800
C	-3.56327500	-4.06078400	-2.20243500
H	-4.57763900	-4.42606300	-2.09437500
C	-0.95203900	-3.10825700	-2.48944100
H	0.04961300	-2.71746000	-2.61465800
C	-1.69922000	-3.40836100	-3.58552200
H	-1.28639500	-3.26480100	-4.57822500
C	-3.02319400	-3.90570100	-3.44052200
H	-3.60413800	-4.15161200	-4.32283500

***(E,Z)*-S_1**

$E(S_0)$ (Ha) = −3390.82423271 Ha

$E(S_1) - E(S_0)$ = 2.09 eV

$E(T_1) - E(S_0)$ = 1.05 eV

C	-0.28775300	-3.44141100	1.35625100
C	-1.59453200	-3.96441400	1.58565500
C	-2.60169900	-3.91714200	0.55483000
C	-3.85021800	-4.56884200	0.80794100
H	-3.77012600	-5.50834600	1.34844000
C	-5.15094400	-4.19472600	0.52163000
C	-6.14656300	-5.20739100	0.71857300
N	-6.95261200	-6.02677200	0.87073600
C	-5.68123300	-2.89185100	0.19058200
C	-6.96427400	-2.61548900	-0.24623100
S	-4.79960400	-1.41090300	0.43474000
C	-7.22639700	-1.24076100	-0.39044700
H	-7.68687100	-3.39209200	-0.46365800

C -6.15379900 -0.44197400 -0.06389100
H -8.16298600 -0.84862200 -0.76500900
C -6.01706900 1.01341500 -0.16656300
C -7.05527300 1.90345100 0.27279700
C -4.86152300 1.54676700 -0.71390900
C -8.22770000 1.47194500 0.94176400
C -6.88583100 3.29855600 0.06557800
C -4.70121300 2.92625100 -0.89021900
H -4.07795100 0.87946500 -1.05457800
C -9.19617900 2.36413900 1.33186600
H -8.35114200 0.42079200 1.16717500
C -7.90023000 4.19830300 0.46863100
C -5.70121900 3.79592100 -0.52402900
H -3.79739300 3.32343300 -1.33663100
C -9.04055100 3.73738300 1.08267600
H -10.08205300 2.00867300 1.84566200
C -7.73973300 5.65094800 0.24406600
C -5.51470200 5.24516700 -0.74735800
H -9.79876700 4.45123000 1.38244000
O -8.59274000 6.46554200 0.55885500
N -6.55762700 6.08191600 -0.35092400
O -4.50114600 5.69993900 -1.25396600
C -6.42291100 7.52204300 -0.55653100
H -7.23670300 7.88354800 -1.18578700
H -6.46910500 8.04026400 0.40203600
H -5.46580300 7.70202100 -1.03577400
C 0.67803800 -3.60271400 2.37037300
H 1.69902400 -3.28853800 2.20205300
C -1.89090900 -4.50049800 2.85863000
H -2.89985300 -4.82392300 3.07882300
C -0.93289900 -4.60650700 3.84417300
H -1.19264900 -5.02909900 4.80830700
C 0.36849000 -4.16874800 3.59072000
H 1.13738200 -4.26521300 4.34912500
C -2.28470300 -3.29202500 -0.69730800
C -1.01881000 -2.65405500 -0.88416400
C 0.03032200 -2.80382100 0.09216200
C 1.35145800 -2.34641300 -0.12170900
H 1.88938000 -2.04025900 0.76964300
C 2.12668100 -2.31056700 -1.28007300
C 1.77182000 -3.05562700 -2.44697800
N 1.54685000 -3.67201200 -3.40300900
C 3.41497200 -1.68423400 -1.33965000
C 4.34476900 -1.74856700 -2.36851000
S 4.02754200 -0.69677500 -0.03762500
C 5.52330100 -1.03713400 -2.10161000
H 4.16273600 -2.29435800 -3.28548600
C 5.52651900 -0.40280600 -0.87508200
H 6.33858000 -0.95264600 -2.80822000
C 6.53435300 0.48177700 -0.29566900
C 7.94126100 0.20419800 -0.40423100
C 6.12742800 1.63218900 0.36453800
C 8.46605300 -0.99685800 -0.94165600
C 8.86280600 1.16278800 0.09671800
C 7.04872300 2.55259600 0.87411400
H 5.06909200 1.85193000 0.44723400
C 9.82055400 -1.20702200 -1.03420300
H 7.78627200 -1.77448700 -1.26375900
C 10.25334800 0.92949700 -0.01769600
C 8.40005500 2.33929700 0.72893000
H 6.71023300 3.45603000 1.36720900
C 10.72410000 -0.23150000 -0.58407400
H 10.19513900 -2.13714000 -1.44612600
C 11.22046300 1.92932300 0.48379000
C 9.34843200 3.34585100 1.24912400
H 11.79478800 -0.38271500 -0.65512700
O 12.42890900 1.78334500 0.38958500
N 10.70660000 3.07400000 1.08531600
O 8.97361300 4.37040800 1.79767600
C 11.68361800 4.04261400 1.57660000
H 12.29517900 4.40540800 0.74965400
H 12.33730100 3.56953000 2.31004300
H 11.13851100 4.86406900 2.03067300
C -3.18190000 -3.31105300 -1.78793700
H -4.08579500 -3.90001000 -1.71388100
C -0.85429100 -1.83823800 -2.02288400
H 0.02718200 -1.21656700 -2.10272400
C -1.79427300 -1.79847500 -3.03231400
H -1.62909500 -1.16952500 -3.89979400
C -2.94371500 -2.58831600 -2.93732100
H -3.66018600 -2.61526600 -3.75062900

(Z,Z)-S_0

$E(S_0)$ (Ha) = −3390.84189529 Ha
$E(S_1) - E(S_0)$ = 3.48 eV
$E(T_1) - E(S_0)$ = 2.11 eV

C 0.06861900 -0.57582600 -1.29310700
C -0.05928400 0.84322800 -1.13410100
C -0.13665300 1.39826300 0.15489400
C -0.21061000 2.87218900 0.30726800
H 0.73229000 3.41043500 0.24011900
C -1.31868000 3.60905900 0.51310900
C -1.12485000 5.03337500 0.64502100
N -0.97522900 6.17498000 0.75313500
C -2.71539600 3.17509700 0.61874700
C -3.77985900 3.98839100 0.91244900
S -3.25109700 1.54488900 0.34479300
C -5.02249200 3.30972300 0.92166200
H -3.67292900 5.04564000 1.12048700
C -4.90869700 1.97638700 0.63325300
H -5.96310000 3.78697900 1.16469100
C -5.95436700 0.93995800 0.60504000
C -7.18371800 1.13444100 -0.10712500
C -5.74829500 -0.24494300 1.28547600
C -7.45796700 2.27628800 -0.90178900
C -8.17222500 0.11690800 -0.04298800

C	-6.72679200	-1.24874100	1.32412400
H	-4.82215400	-0.39139300	1.82955200
C	-8.65570200	2.41616900	-1.55764300
H	-6.70051300	3.04274200	-1.00276700
C	-9.40188900	0.28622400	-0.72141700
C	-7.92842200	-1.07137900	0.68301700
H	-6.55433700	-2.16691200	1.87291900
C	-9.64200100	1.42016900	-1.45993100
H	-8.84067300	3.29659700	-2.16244100
C	-10.44201800	-0.76262800	-0.65057500
C	-8.95142000	-2.13756000	0.75901400
H	-10.59256600	1.52113400	-1.97038300
O	-11.52333000	-0.65864800	-1.20705800
N	-10.15078900	-1.90720100	0.08644500
O	-8.76554400	-3.17279400	1.37820000
C	-11.19433700	-2.92850000	0.13660700
H	-12.10262700	-2.50858000	0.56986700
H	-11.42021600	-3.27821800	-0.87138100
H	-10.82876700	-3.74841800	0.74667600
C	0.12274500	-1.10972100	-2.61949800
H	0.20924600	-2.18231500	-2.74610800
C	-0.11494200	1.65937700	-2.30811500
H	-0.21541900	2.73247000	-2.19230900
C	-0.05082500	1.11102400	-3.55160800
H	-0.09365700	1.74688000	-4.42929100
C	0.06644800	-0.29730600	-3.70951400
H	0.10868500	-0.72262600	-4.70641800
C	-0.06860200	0.57579800	1.29303700
C	0.05930100	-0.84325700	1.13403100
C	0.13666700	-1.39829100	-0.15496300
C	0.21062300	-2.87221700	-0.30734400
H	-0.73228100	-3.41046100	-0.24023200
C	1.31869800	-3.60908800	-0.51315400
C	1.12487200	-5.03340400	-0.64507100
N	0.97524800	-6.17500700	-0.75319600
C	2.71541500	-3.17512300	-0.61875700
C	3.77989100	-3.98841600	-0.91241700
S	3.25109700	-1.54490500	-0.34482700
C	5.02251900	-3.30974000	-0.92161400
H	3.67297300	-5.04567000	-1.12043600
C	4.90870800	-1.97639800	-0.63323900
H	5.96313500	-3.78699500	-1.16461000
C	5.95436900	-0.93996100	-0.60502200
C	7.18370900	-1.13442300	0.10716900
C	5.74830200	0.24492700	-1.28548200
C	7.45795100	-2.27625400	0.90185800
C	8.17221000	-0.11688500	0.04303100
C	6.72679300	1.24873100	-1.32413000
H	4.82217000	0.39136200	-1.82957900
C	8.65567600	-2.41611600	1.55773500
H	6.70050100	-3.04271100	1.00283700
C	9.40186400	-0.28618100	0.72148400
C	7.92841200	1.07138800	-0.68299900
H	6.55434200	2.16689100	-1.87294300
C	9.64197100	-1.42011200	1.46002200
H	8.84064200	-3.29653200	2.16255200
C	10.44198800	0.76267500	0.65064100
C	8.95140500	2.13757400	-0.75899600
H	10.59252800	-1.52106200	1.97049300
O	11.52329300	0.65871100	1.20714200
N	10.15076400	1.90723500	-0.08640200
O	8.76553300	3.17279600	-1.37820300
C	11.19430600	2.92853900	-0.13656400
H	12.10260400	2.50862000	-0.56981100
H	11.42017300	3.27826800	0.87142300
H	10.82873800	3.74844900	-0.74664600
C	-0.12272300	1.10969200	2.61942900
H	-0.20921900	2.18228600	2.74604000
C	0.11495700	-1.65940700	2.30804500
H	0.21543000	-2.73249900	2.19223900
C	0.05084300	-1.11105300	3.55153900
H	0.09367500	-1.74691000	4.42922200
C	-0.06642500	0.29727700	3.70944500
H	-0.10865900	0.72259700	4.70635000

(Z,Z)-S$_1$

$E(S_0)$ (Ha) = −3390.82148596 Ha

$E(S_1) - E(S_0)$ = 2.11 eV

$E(T_1) - E(S_0)$ = 1.08 eV

C	0.72235800	-0.55415000	-1.10947400
C	0.38567200	0.83257200	-1.09756900
C	-0.37084700	1.40040300	-0.00624700
C	-0.66903700	2.79223400	-0.05008300
H	0.11645800	3.41544500	-0.46799700
C	-1.80803200	3.50141900	0.30977200
C	-1.65779200	4.92559000	0.36199100
N	-1.53404300	6.07756100	0.40993700
C	-3.15759000	3.03573200	0.49517400
C	-4.22348800	3.77879200	0.98070300
S	-3.70828200	1.46286800	-0.01157900
C	-5.44779600	3.09337600	0.94926700
H	-4.10724200	4.78777900	1.35598500
C	-5.34946300	1.81551700	0.43913600
H	-6.37196300	3.50695700	1.33117600
C	-6.37919300	0.78192700	0.32866300
C	-7.70845400	1.08143600	-0.12843700
C	-6.07033500	-0.52107900	0.68789800
C	-8.10072900	2.34833600	-0.62702200
C	-8.67738500	0.04238000	-0.12468200
C	-7.03148800	-1.53753400	0.66342900
H	-5.07164100	-0.75219200	1.04051800
C	-9.38875400	2.58531300	-1.04135600
H	-7.36502500	3.13808300	-0.70340600
C	-9.99991700	0.31142100	-0.54831700
C	-8.32402500	-1.26484800	0.28081700
H	-6.77576900	-2.54578100	0.96674100

C	-10.35193900	1.56505400	-0.98939100
H	-9.66168200	3.56278900	-1.42237400
C	-11.01945600	-0.75957400	-0.53364600
C	-9.32364200	-2.35338900	0.29173000
H	-11.37224100	1.73984400	-1.30988200
O	-12.17713200	-0.57307400	-0.87332400
N	-10.61694700	-2.02349400	-0.11280900
O	-9.04288600	-3.49054500	0.63656000
C	-11.64258300	-3.06386000	-0.11311100
H	-12.46612400	-2.77196200	0.53926400
H	-12.03163100	-3.20150600	-1.12263000
H	-11.18580900	-3.98192100	0.24268500
C	1.28706200	-1.09239600	-2.28534600
H	1.46307700	-2.15719600	-2.34392800
C	0.70006400	1.60566400	-2.23778800
H	0.41528000	2.64875700	-2.27165000
C	1.32170800	1.06231800	-3.34117800
H	1.55197400	1.69014800	-4.19469700
C	1.60215100	-0.30633300	-3.37235500
H	2.04240100	-0.75679600	-4.25489900
C	-0.72232600	0.55369400	1.10904100
C	-0.38564800	-0.83303400	1.09713300
C	0.37092300	-1.40085300	0.00583300
C	0.66923000	-2.79264900	0.04968400
H	-0.11619000	-3.41594300	0.46761200
C	1.80829600	-3.50171400	-0.31020700
C	1.65819400	-4.92589700	-0.36249100
N	1.53454300	-6.07787200	-0.41059200
C	3.15780700	-3.03588500	-0.49554800
C	4.22379000	-3.77879500	-0.98113200
S	3.70833800	-1.46301900	0.01137500
C	5.44803500	-3.09327800	-0.94956600
H	4.10764700	-4.78775100	-1.35653000
C	5.34957100	-1.81548200	-0.43929500
H	6.37225500	-3.50673800	-1.33147800
C	6.37920700	-0.78182300	-0.32862900
C	7.70846800	-1.08127700	0.12850700
C	6.07025400	0.52120800	-0.68769700
C	8.10082800	-2.34821400	0.62693200
C	8.67730300	-0.04213000	0.12497100
C	7.03131000	1.53774800	-0.66301600
H	5.07156100	0.75228200	-1.04034500
C	9.38884800	-2.58513200	1.04131700
H	7.36519200	-3.13804000	0.70315300
C	9.99983100	-0.31110900	0.54865900
C	8.32384700	1.26512600	-0.28035500
H	6.77551600	2.54601600	-0.96619200
C	10.35194000	-1.56477500	0.98956900
H	9.66184100	-3.56263900	1.42220900
C	11.01926800	0.75998600	0.53422400
C	9.32335800	2.35376600	-0.29102200
H	11.37223600	-1.73951800	1.31010500
O	12.17693900	0.57354500	0.87395400
N	10.61666800	2.02393100	0.11355100
O	9.04252000	3.49094800	-0.63570000
C	11.64220500	3.06439500	0.11407900
H	12.46582000	2.77266900	-0.53827900
H	12.03116700	3.20193200	1.12364700
H	11.18536900	3.98246300	-0.24161800
C	-1.28707800	1.09192500	2.28489800
H	-1.46309000	2.15672400	2.34349300
C	-0.70009400	-1.60613500	2.23732900
H	-0.41531700	-2.64923000	2.27119800
C	-1.32178700	-1.06280200	3.34069700
H	-1.55209500	-1.69064300	4.19419700
C	-1.60222200	0.30585000	3.37188100
H	-2.04251600	0.75630300	4.25440800

5. References

[1] O. K. Kwon, J.-H. Park, S. K. Park, S. Y. Park, *Adv. Energy Mater.* **2015**, *5*, 1400929.

[2] J. Shi, A. Isakova, A. Abudulimu, M. van den Berg, O. K. Kwon, A. J. Meixner, S. Y. Park, D. Zhang, J. Gierschner, L. Lüer, *Energy Environ. Sci.* **2018**, *11*, 211–220.

[3] S. S. Murali, J. K. Gallaher, C. Janiseck, E. J. Tay, I. Wagner, K. E. Thorn, A. Ilina, R. R. Tamming, J. Wang, C. Sester, J. J. Sutton, M. B. Price, K. C. Gordon, K. Chen, X. Zhan, J. M. Hodgkiss, P. A. Hume, *J. Am. Chem. Soc.* **2023**, *145*, 732–744.

[4] J. C. De Mello, H. F. Wittmann, R. H. Friend, *Adv. Mater.* **1997**, *9*, 230–232.

[5] T. Sato, Y. Hamada, M. Sumikawa, S. Araki, H. Yamamoto, *Ind. Eng. Chem. Res.* **2014**, *53*, 19331–19337.

[6] J. Jaumot, R. Gargallo, A. De Juan, R. Tauler, *Chemometr. Intell. Lab. Syst.* **2005**, *76*, 101–110.

[7] K. Chen, J. K. Gallaher, A. J. Barker, J. M. Hodgkiss, *J. Phys. Chem. Lett.* **2014**, *5*, 1732–1737.

[8] Gaussian 16, Revision C.01, M. J. Frisch, G. W. Trucks, H. B. Schlegel, G. E. Scuseria, M. A. Robb, J. R. Cheeseman, G. Scalmani, V. Barone, G. A. Petersson, H. Nakatsuji, X. Li, M. Caricato, A. V. Marenich, J. Bloino, B. G. Janesko, R. Gomperts, B. Mennucci, H. P. Hratchian, J. V. Ortiz, A. F. Izmaylov, J. L. Sonnenberg, D. Williams-Young, F. Ding, F. Lipparini, F. Egidi, J. Goings, B. Peng, A. Petrone, T. Henderson, D. Ranasinghe, V. G. Zakrzewski, J. Gao, N. Rega, G. Zheng, W. Liang, M. Hada, M. Ehara, K. Toyota, R. Fukuda, J. Hasegawa, M. Ishida, T. Nakajima, Y. Honda, O. Kitao, H. Nakai, T. Vreven, K. Throssell, J. A. Montgomery, Jr., J. E. Peralta, F. Ogliaro, M. J. Bearpark, J. J. Heyd, E. N. Brothers, K. N. Kudin, V. N. Staroverov, T. A. Keith, R. Kobayashi, J. Normand, K. Raghavachari, A. P. Rendell, J. C. Burant, S. S. Iyengar, J. Tomasi, M. Cossi, J. M. Millam, M. Klene, C. Adamo, R. Cammi, J. W. Ochterski, R. L. Martin, K. Morokuma, O. Farkas, J. B. Foresman, and D. J. Fox, Gaussian, Inc., Wallingford CT, **2016**.

[9] T. Yanai, D. P. Tew, N. C. Handy, *Chem. Phys. Lett.* **2004**, *393*, 51–57.
